# Antidiabetic Effect of *Rehmanniae* Radix Based on Regulation of TRPV1 and SCD1

**DOI:** 10.3389/fphar.2022.875014

**Published:** 2022-05-26

**Authors:** Ye Liu, Ruizheng Zhu, Bei Liu, Wuqing Wang, Ping Yang, Zhonglian Cao, Xiaolei Yang, Wandi Du, Qing Yang, Jingru Liang, Jiarong Hu, Guo Ma

**Affiliations:** ^1^ School of Pharmacy, Fudan University, Shanghai, China; ^2^ Department of Dermatology, Minhang Hospital, Fudan University, Shanghai, China; ^3^ Ruijin Hospital, Shanghai Jiao Tong University School of Medicine, Shanghai, China

**Keywords:** *Rehmanniae* Radix, diabetes mellitus, transient receptor potential vanilloid 1, stearoyl-CoA desaturase 1, network pharmacology, transcriptome

## Abstract

**Purpose:** This study aimed to disclose the antidiabetic mechanisms of *Rehmanniae* Radix (RR).

**Methods:** The antidiabetic effect of RR was studied in Streptozocin (STZ)–induced diabetes mellitus (DM) rats and HepG2 cells with insulin resistance (IR). Antidiabetic targets and signaling pathways of RR were confirmed by the network pharmacology and transcriptome analysis as well as HK2 cells induced by high glucose (HG).

**Results:** After the DM rats were administrated RR extract (RRE) for 4 weeks, their body weight was 10.70 ± 2.00% higher than those in the model group, and the fasting blood glucose (FBG), AUC of the oral glucose tolerance test, and insulin sensitivity test values were 73.23 ± 3.33%, 12.31 ± 2.29%, and 13.61 ± 5.60% lower in the RRE group, respectively. When compared with the model group, an increase of 45.76 ± 3.03% in the glucose uptake of HepG2 cells with IR was seen in the RRE group. The drug (RR)–components–disease (DM)–targets network with 18 components and 58 targets was established. 331 differentially expressed genes (DEGs) were identified. TRPV1 and SCD1 were important DEGs by the intersectional analysis of network pharmacology and renal transcriptome. The *TRPV1* overexpression significantly inhibited apoptosis and oxidative stress of the HK2 cells induced by HG, while *SCD1* overexpression induced apoptosis and oxidative stress of the HK2 cells induced by low and high glucose. When compared to the HG group, the mRNA and protein expressions of TRPV1 in the presence of RRE (100 μg/ml) increased by 3.94 ± 0.08 and 2.83 ± 0.40 folds, respectively.

**Conclusion:** In summary, RR displayed an inspiring antidiabetic effect by reducing FBG and IR, upregulating the mRNA and protein expressions of TRPV1, and downregulating mRNA expression of SCD1. Induction of TRPV1 and inhibition of SCD1 by RR was possibly one of its antidiabetic mechanisms.

## Introduction

Diabetes mellitus (DM) is a metabolic/endocrine disorder with an increasing incidence and prevalence worldwide. According to the diabetes atlas of the International Diabetes Federation (IDF Diabetes Atlas 10th edition), 537 million adults (20–79 years) suffer from DM. This number is predicted to rise to 643 million by 2030 and 783 million by 2045. A large national survey (Li et al., 2020) indicated that the total number of patients with DM in the Chinese mainland is estimated to be 129.8 million. DM is characterized by hyperglycemia caused directly by insufficient insulin secretion or insulin resistance (IR). Its pathology is irreversible and is often accompanied by many complications. The blood vessels, heart, brain, kidney, nerves, eyes, feet, and other organs are damaged by long-term hyperglycemia, leading to death in severe cases (DiMeglio et al., 2018; Lascar et al., 2018).

Herbs [e.g., *Rehmanniae radix* (Gaert.) Libosch, *Astragalus membranaceus* (Fisch.) Bunge, *Ginkgo biloba* Linn, *Pueraria lobata* (Willd.) Ohwi] have been used to treat DM for thousands of years, those which exhibit favorable antidiabetic effects and low side effects (Yokozawa et al., 1986; Shi et al., 2011; Tong et al., 2012; Wen et al., 2012; Tzeng et al., 2013). As one of the most commonly used antidiabetic herbs, RR has been recorded in Chinese pharmacopoeia and many classical prescriptions such as Liuwei Dihuang decoction, Huanglian Dihuang decoction, and Dangguiliuhuang decoction (Zhang et al., 2008; Cao et al., 2017). *Rehmanniae* Radix (RR) displayed favorable clinical effects in the treatment of metabolic diseases such as DM (Kim et al., 2017). RR and its components, e.g., iridoid glycoside, catalpol, rehmannioside D, oligosaccharide, and polysaccharide, have been proven to ameliorate DM by improving IR, regulating lipid metabolism, protecting islet *ß* cells, and anti-inflammatory and antioxidant (Kitagawa et al., 1971; Zhang et al., 2008; Choi et al., 2013; Zhang et al., 2013; Zhang et al., 2014; Yan et al., 2018).

As everyone knows, herbs possess multicomponent and multi-target characteristics, which makes research on the antidiabetic mechanism of herbs full of challenges. In 2007, Hopkins (2007) proposed the theory of network pharmacology, believing that drugs produce pharmacodynamics and toxic effects based on the interaction of drug–disease–target (Hopkins, 2007, 2008), which coincides with the core idea of the Traditional Chinese medicine (TCM) theory. Network pharmacology provides an opportunity to forecast and explain the mechanism and pharmacodynamic material of antidiabetic herbs. Network pharmacology has been widely used to study the antidiabetic mechanism of multiple herbs and their preparations, e.g., *Coptis chinensis* Franch, *Hedysarum multijugum* Maxim, *Sorghum bicolor* (Linn.) Moench (L.), leaf of *Morus alba* Linn (Xu et al., 2020; Xu et al., 2020c; Guo et al., 2020; Meng et al., 2020; Oh et al., 2020; Pan et al., 2020; Wu and Hu, 2021). At present, there are few studies on RR against DM through network pharmacology.

As a macro approach, transcriptomics has changed the research mode of a single gene and brought omics research into the era of rapid development (Lander et al., 2001; Zhao et al., 2018). The application of transcriptomics in the field of herbs helps in explaining the syndrome differentiation theory of TCM and the action mechanism of herbs at the gene level. The antidiabetic targets and signaling pathways (e.g., PI3K/Akt/FoxO1 and PPARɑ) of herbs such as *Coptis chinensis* Franch., *Panax ginseng* C. A. Meyer, *Alisma orientale*, and *Potentilla discolor* Bunge have been studied, identified, and validated by transcriptome (Han et al., 2019; Xu et al., 2020b; Yang et al., 2020; Wu et al., 2021).

At present, some targets and signaling pathways of RR and its component catalpol against DM have been revealed, such as AGEs/RAGE/SphK1, Nrf-2, mitogen-activated protein kinases (MAPK), nuclear localization of nuclear factor kappa B (NF-ΚB), and the AMPKX4/PI3K/AKT signaling pathway (Choi et al., 2013; Lv et al., 2016; Mohamed and Samak, 2017; Yan et al., 2018).

Among the numerous antidiabetic targets, transient receptor potential vanilloid 1 (TRPV1) and stearoyl-CoA desaturase 1 (SCD1) were particularly interesting. TRPV1 was discovered by David Julius in 1997 and was identified as an ion channel that can be activated by heat and capsaicin to cause pain, for which David Julius was awarded the 2021 Nobel Prize. In recent years, numerous studies have demonstrated that TRPV1 plays a vital role in metabolic diseases (Radu et al., 2013). TRPV1 is linked to adipose accumulation and energy expenditure by inhibiting the development of preadipocytes into mature adipocytes and increasing their lipolysis (Zhang et al., 2007). It has been proven that TRPV1 activation may mediate the browning of white adipose tissue (WAT) *via* activating peroxisome proliferators–activated receptors γ (PPARγ), positive regulatory domain containing 16 (PRDM16), and the AMPK signaling pathway (Baskaran et al., 2016), which indicates that TRPV1 is a promising target against DM.

SCD1 is a rate-limiting enzyme that converts saturated fatty acids (SFA) to monounsaturated fatty acids (MUFA). It plays an important role in metabolic abnormalities (Liew et al., 2004; Liu et al., 2013) by affecting insulin sensitivity and lipid metabolism (Popeijus et al., 2008; Sampath and Ntambi, 2011). It is a potential therapeutic target for DM, obesity, hypertension, and dyslipidemia (Enoch et al., 1976).

In this study, the DM rats were constructed to study the antidiabetic efficacy and mechanism of RR. The HepG2 cells with IR were established to study the influence of RR on glucose uptake. The drug (RR)–components–disease (DM)–targets network was established, and the antidiabetic targets and signaling pathways of RR were identified by the combination of network pharmacology and transcriptomics analysis. The role of TRPV1 and SCD1 in the treatment of DM was studied using the HK2 cells with the overexpressions of the two key targets in the low- and high-glucose groups, respectively. Regulation of mRNA and protein expressions of TRPV1 and SCD1 by RRE was assayed by qRT-PCR and western blot. This study will be conducive to disclosing the antidiabetic mechanism of RR from multiple perspectives, especially the regulation of TRPV1 and SCD1.

## Materials and Methods

### Chemicals and Biological Products

*Rehmanniae* Radix extract (30:1, i.e., 1 g extract/30 g herb) was purchased from Xi’an Xinlu Biotechnology Co., Ltd. (#20200910, China). Streptozocin (STZ, #S0130), recombinant human insulin (#91077C), and metformin hydrochloride (PHR1084) were obtained from Sigma-Aldrich Inc. (United States). 2-[N-(7-nitrobenz-2-oxa-1,3-diazol-4-yl)amino]-2-deoxyglucose (2-NBDG, #GC10289) was obtained from GlpBio Inc. (United States). Insulin aspart 30 injection (#S20133006) was obtained from Novartis Inc. (Switzerland). cDNA Synthesis SuperMix for qPCR (#11141ES6), qPCR SYBR Green Master Mix (#011199ES08), Annexin V-Alexa Fluor 647/PI Apoptosis Detection Kit (#40304ES60), JC-1 Mitochondrial Membrane Potential Assay Kit (#40706ES60), Mitochondrial ROS Detection Kit (#50102ES02), and CCK*-*8 assay kit (#40203ES80) were obtained from Yeasen Co., LTD. (China). Lipofectamine 3000 (#L3000075) was obtained from Thermo Scientific (United States). The TRPV1, SCD1, and GAPDH antibodies were obtained from Cell Signaling Technology (United States). The HepG2 and HK2 cells were purchased from ATCC (American Type Culture Collection, United States).

### Cell Culture

The human hepatocellular carcinoma cell line (HepG2) was cultured in Dulbecco’s modified Eagle’s medium (DMEM) with 25.5 mM glucose, 1% penicillin/streptomycin, and 10% fetal bovine serum under 5% CO_2_ and 95% air at 37°C. The human renal proximal tubular epithelial cell line (HK2) was cultured in DMEM with 5.5 mM glucose, and all the other conditions remained the same as for HepG2. When the cells grow to about 80% fusion, the passage was carried out in a ratio of 1:2.

### Cell Viability

The cells were seeded in 96-well plates at a density of 5 × 10^4^ cells per milliliter. After 24-h culture, the cells were incubated with different stimulation for 48 h. Then, the cell viability was evaluated using the CCK-8 kit (Meilunbio, China) following the manufacturer’s instructions. The OD value was detected at 450 nm by a microplate reader. The cell survival rate was calculated according to the following formula:

$$Cell\;survival\;rate\;=\;\left[\frac{As\;-\;Ab}{Ac\;-\;Ab}\right]\times 100\%,$$

where A_s_ is the absorbance of the experimental well (given stimulation); A_c_ is the absorbance of the control wells (no stimulation); and A_b_ is the absorbance of the blank well (no cells).

### Glucose Uptake

The HepG2 cells were cultured in 12-well plates at a density of 5 × 10^4^ cells per milliliter. The optimal concentration (25, 50, 100, 200, and 300 μM) of 2-NBDG (E_x_/E_m_ = 502/530 nm) was explored. The cells were incubated with 2-NBDG solution diluted in glucose-free DMEM for 1 h, with three duplicates at each concentration. After PBS washing, the cells were digested and suspended again and detected within 1 h. Fluorescence was detected by the FITC channel from flow cytometry (FCM), which was characterized by the mean area of FITC, and the results were analyzed by the FlowJo software.

### Influence of *Rehmanniae* Radix Extract on HepG2 Cells With Insulin Resistance

The HepG2 cells were cultured in 12-well plates at a density of 5 × 10^4^ cells per milliliter. A high concentration of insulin was used to induce insulin resistance in the HepG2 cells. The HepG2 cells were incubated with a series of insulin solutions (0.5, 5, 10, 20, and 40 μg/ml) for 48 h to determine the optimal insulin concentration so as to reach the lowest glucose uptake. To investigate the influence of RRE on the HepG2 cells with IR, the blank control group [insulin (−), drug (−)], the model group [insulin (+), RRE (−)], the positive control group [insulin (+), 1 mM metformin], and the administration group [insulin (+), 25, 50, 100 μg/ml of RRE] were incubated with the HepG2 cells for 48 h. The assays were performed in triplicates. Then, the glucose uptake detection was performed.

### Animals

The rats were reared in the Laboratory Animal Center, School of Pharmacy, Fudan University. The temperature was set at 25 ± 2°C, the humidity was kept at 45–50%. All the animal cages, feed, drinking water, and bedding materials were sterilized by high-pressure steam. All the laboratory operations were conducted in an ultraclean platform. After 2 weeks of adaptive feeding, 15 healthy Sprague Dawley (SD) male rats (200–250 g) were randomized into groups and fasted for 12 h, from which 10 rats were intraperitoneally injected with 1% STZ solution at a dose of 60 mg/kg bodyweight, and an equal volume of saline was intraperitoneally injected into the other five rats. On the 7th day after injection, all the rats were fasted for 12 h, and the fasting blood glucose (FBG) level was tested. When the FBG of the rats was ≥16.7 mmol/L (300 mg/dl), it was considered to meet the standards of DM. The DM rats were randomly divided into the model group (*n* = 5) and RRE group (*n* = 5), and the healthy rats (*n* = 5) were divided into the normal group. The normal group and model group were given an intragastric administration of saline once a day, and the RRE group was given RRE suspension at the dose of 450 mg/kg once a day. The administration lasted for 4 weeks, and the fasting body weight and FBG of all the rats were measured weekly. One week after the last experiment, the kidneys, small intestines, and skeletal muscles of the rats were collected and stored at −80°C. All animal care and experimental protocols complied with the Animal Management Rules of the Ministry of Health of the People’s Republic of China and were approved by the Animal Ethics Committee of the School of Pharmacy, Fudan University (Ethics approval No. 2020-04-LY-MG-01, Shanghai, China).

### Oral Glucose Tolerance Test and Insulin Sensitivity Test

An oral glucose tolerance test (OGTT) was performed 4 weeks after the administration of RRE. After fasting for 12 h, all the rats were weighed and their FBG was detected. Glucose solution was orally administrated to the rats at the dose of 2 g/kg body weight. Blood glucose was detected at 30, 60, 120, 180, and 240 min after administration. An insulin sensitivity test (IST) was performed a week after the OGTT. After fasting for 12 h, all the rats were weighed and their FBG were detected. The solution of insulin was injected intraperitoneally into the rats at a dose of 0.8 U/kg body weight. Blood glucose was detected at 30, 60, 120, and 180 min after insulin administration.

### Network Pharmacology of *Rehmanniae* Radix

#### Active Components Screening

All the components of RR were obtained from a systematic pharmacological database of TCM, i.e., TCMSP (https://old.tcmsp-e.com/tcmsp.php). Two ways to filter these components were conducted, one of which was the conditioning of the TCMSP database, which was set at an oral absorption rate (OB) ≥20% and druglikeness (DL) ≥0.1. The second way was the ADME filtering through the SwissADME database (http://www.swissadme.ch/), which included absorption, distribution, metabolism, and excretion. The relevant parameters were calculated by the database when the structural formula of the components was input. The components were included when GI absorption in the pharmacokinetics parameter was high and at least two “yes” appeared in the five parameters of druglikeness (Lipinski, Ghose, Veber, Egan, and Muegge). The components included in the TCMSP and Swiss databases were combined.

#### Prediction and Intersection of Target

The TCMSP and Swiss databases were used to predict drug-related targets. The protein name of targets obtained from the TCMSP database was converted into the corresponding gene name by the UniProt database (https://www.uniprot.org/). The targets were also predicted using the SwissTargetPrediction database (http://www.swisstargetprediction.ch/) by entering the component structure formula and were screened according to a probability *p* value >0.4. The drug-related targets were obtained by merging the two databases, of which the repeated targets of the same component were removed. For the disease-related targets, the GeneCards database (http://www.genecards.org/) was searched using “Diabetes” or “diabetic” as the keywords. Venny 2.1.0 tool (https://bioinfogp.cnb.csic.es/tools/venny/index.html)was used to obtain the intersection of the drug-related targets and disease-related targets.

#### Protein–Protein Interaction Network Construction

Calculation of the intersection targets was performed by the String database platform (https://www.string-db.org/). The conditions were set to *Homo sapiens*, and the minimum required interaction score to ≥0.4. Proteins without interaction were removed. The number of nodes connected by each target was calculated and sorted by R language.

### Transcriptome Analysis

The kidney, small intestine, and skeletal muscle tissue samples from the rats (three in each group) were used for the transcriptome analysis by Shanghai Majorbio Bio-pharmTechnology Co., Ltd, using the Illumina HiSeq™ 2500 sequencer. The raw data obtained by the Illumina sequencing platform were analyzed following the process of sequence alignment, transcript assembly, functional annotation and query, and expression analysis. Transcripts per million (TPM) reads were used to characterize gene expression, and the calculation formula is as follows:

$$TPM=\frac{R\times 10^{6}}{\left(\frac{R_{1}}{l_{1}}+\frac{R_{2}}{l_{2}}+\_+\frac{R_{n}}{l_{n}}\right)\times l},$$

where R and L represent the read counts and gene lengths of the genes to be calculated, respectively. R_1_–R_n_ and L_1_–L_n_ represent the read counts and gene lengths of all genes in the sample, respectively.

The DESeq2 software was used to calculate the differential expression of genes according to the TPM value. Differentially expressed genes (DEGs) were identified with the standard of |FC| >1.5 and *p* value <0.05. The DEGs were intersected and divided into two parts, i.e., upregulation and downregulation, and the 10 DEGs with the lowest *p* values were selected to verify the gene expression trend by qRT-PCR.

### Gene Ontology and Kyoto Encyclopedia of Genes and Genomes Analyses

GO and KEGG enrichment analyses were performed on the DAVID database (https://david.ncifcrf.gov/). The GO terms mainly included molecular functions, cellular components, and biological processes. The KEGG pathway was classified according to the Kyoto Encyclopedia of Genes and Genomes (KEGG) database (https://www.kegg.jp/kegg/pathway.html). When p.adjust was <0.1, the GO terms and KEGG pathways were considered to be significantly enriched.

### Quantitative Real-Time Polymerase Chain Reaction

Total RNA of the rat kidney tissues or HK2 cells was isolated using RNAiso Plus (TaKaRa Biotech, China). After reverse-transcription, qRT-PCR was performed by the Applied Biosystems QuantStudio 3 system (Thermo Fisher, American). The reactions were performed following the cycling conditions: 95°C for 10 s, followed by 40 cycles of 95°C for 5 s, 60°C for 30 s, and 72°C for 30 s. The mRNA level of the target genes was normalized and analyzed by the 2^−ΔΔCT^ method. The primers were designed from the Prime-BLAST function of the National Center for Biotechnology Information (NCBI) database and synthesized by Shenggong Bioengineering Co., Ltd. (Shanghai, China). The primer sequences are listed in Supplementary Table S2.

### Cell Transfection

The coding sequences of *TRPV1* and *SCD1* were constructed on pcDNA3.1-3× Flag (+) vector by Hunan Fenghui Biotechnology Co., Ltd. (Fenghui, China). The plasmid extraction was carried out according to the plasmid extraction kit scheme (# DP103-03, Tiangen Biotech, China). The HK2 cells were cultured in 12-well plates at a density of 5 × 10^4^ cells per milliliter for 24 h. Transfection of *TRPV1* and *SCD1* as well as the vector in HK2 cells (1,250 ng plasmids per well) was performed using Lipofectamine^®^ 3000 (#L3000015, Thermo Fisher Scientific, United States). And every transfection group was divided into low-glucose (5.5 mM) and high-glucose (60 mM) groups with three duplicates. Total RNA was extracted 48 h after transfection, and the gene overexpression was verified by qRT-PCR.

### Cell Apoptosis Detection

The HK2 cells transfection was carried out as described above. Apoptosis was evaluated using the Annexin V-Alexa Fluor 647/Propidium Iodide (PI) Apoptosis Detection Kit 48 h after transfection. The cells were digested by EDTA-free trypsin, washed with PBS, and resuspended with the binding buffer. The cells in each well were incubated with Annexin V-Alexa Fluor 647 (5 μl) and PI (10 μl) at room temperature in the dark for 15 min. The detection was conducted by using the APC and PE channels from FCM within 1 h.

### Reactive Oxygen Species Detection

The HK2 cells transfection was carried out as described above. DCFH-DA (10 mM) was diluted with serum-free low-glucose DMEM at a ratio of 1:3000 to obtain the staining solution. 500 μl of the staining solution was added into each well and incubated at 37°C for 30 min. After the probe was transferred, the cells were washed with PBS, digested and resuspended, and detected by FCM within 1 h. The fluorescence intensity of the cells was measured by the FITC channel.

### Mitochondrial Superoxide Detection

The HK2 cells transfection was carried out as described above. Mitochondrial superoxide (MitoSOX, 5 mM) was diluted with serum-free low-glucose DMEM at a ratio of 1:1,000 to obtain the staining solution. Probe loading was carried out the same as " ROS detection," but the incubation time was 20 min. The cells were digested and resuspended, and then detected by the PI channel of FCM.

### Mitochondrial Membrane Potential Detection

The HK2 cells transfection was carried out as aforementioned. The JC1 staining working solution and buffer solution were configured according to the instruction of the kit. The probe loading and cell collection methods were the same as 1.14. The cells were detected by FCM within 1 h. The fluorescence intensity of the cells was measured by FITC and PE double-channel.

### Western Blot

Proteins from the HK2 cells were extracted by using RIPA buffer with 1% of PMSF and protease inhibitor cocktail following the standard protocol, and then protein concentrations were quantified using a bicinchoninic acid (BCA) protein assay kit. Equal amounts of protein extracts were loaded on SDS-PAGE (150 V, 80 min) and electrotransferred to a 0.45-μm PVDF membrane (200 mA, 90 min). The membranes were blocked with 5% nonfat milk for 1 h at room temperature and incubated with primary antibodies overnight at 4°C. On the next day, fluorescence-conjugated secondary antibodies were applied to the membranes for 1 h at room temperature. The membranes were visualized with FluorChem Alpha Imaging (ProteinSimple, United States). Target proteins were normalized to GAPDH and quantified by the ImageJ software.

### Effect of *Rehmanniae* Radix Extract on mRNA and Protein Expressions of TRPV1 and SCD1

The HK2 cells were seeded at a density of 5 × 10^4^/ml in 6-well plates. The cells were grouped into low-glucose (LG) and high-glucose (HG) groups; and the HG groups were further divided into groups of RRE administration (25, 100, and 400 μg/ml) and positive control (CAP, capsaicin, 10 μM). Total RNA and protein were extracted after 48 h of the administration, and the mRNA and protein expressions of TRPV1 and SCD1 were detected by qRT-PCR and Western blot, respectively.

### Statistical Analysis

The data were analyzed by GraphPad Prism 9.0 software for Windows (GraphPad Software, San Diego, CA). Two-way ANOVA and one-way ANOVA were used. *p* < 0.05 was considered to be significant.

## Results

### Cytotoxicity of *Rehmanniae* Radix Extract and Glucose

RRE was nontoxic to the HepG2 and HK2 cells within the concentration range of 0–400 μg/ml, and the cell survival rate was above 80% (Supplementary Figure S1A). Moreover, the survival rate of the HK2 cells increased with an increase of RRE concentration and decreased with an increase of glucose concentration (5.5–120 mM) (Supplementary Figure S1B). The survival rate of the HK2 cells was lower than 80% at 80 mM of glucose (Supplementary Figure S1C). Finally, 60 mM was chosen as the concentration for high-glucose stimulation.

### *Rehmanniae* Radix Extract Increases Glucose Uptake of HepG2 Cells With Insulin Resistance

**FIGURE 1:**
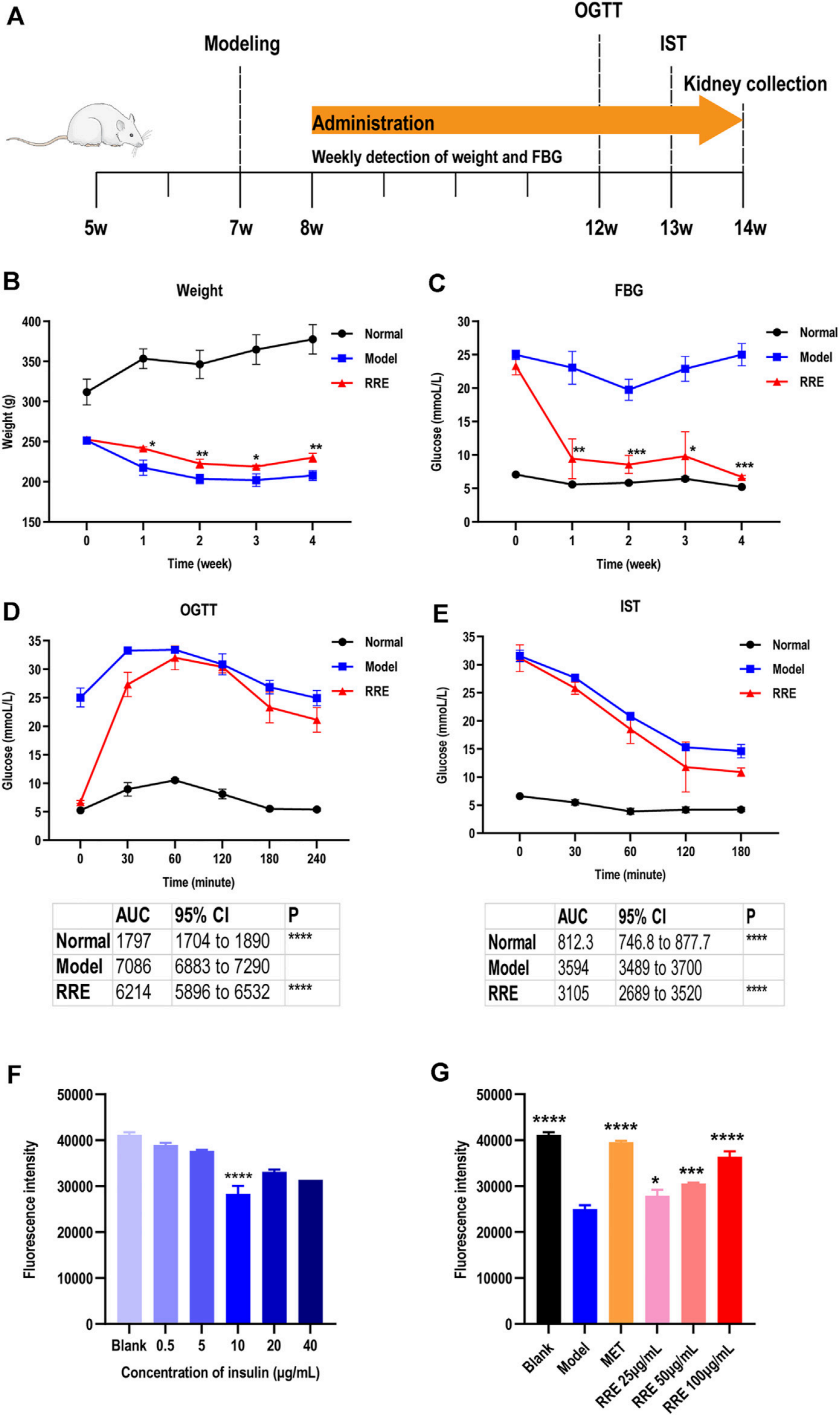
Antidiabetic effect of RRE in *in vivo* and *in vitro* studies. **(A)** Schematic timetable for the suggested antidiabetic experiments *in vivo*. The SD rats were induced by STZ at 7 weeks of age. After 4 weeks of RRE administration, the metabolic phenotype of the rats was evaluated by an oral glucose tolerance test (OGTT) at 12 weeks of age, followed by 1 week of recovery time and subsequently an IST. **(B)** Weekly weight of the SD rats in different groups. **(C)** Weekly FBG of the SD rats in different groups. **(D)** Blood glucose of the SD rats at each time point in the OGTT. The table shows the area under the curve (AUC) and its 95% CI. **(E)** Blood glucose at each time point in the insulin sensitivity test. The table shows the area under the curve (AUC) and its 95% CI. **(F)** Influence of different concentrations of insulin on glucose uptake in HepG2 cells. Glucose uptake was characterized by fluorescence intensity of 2-NBDG. ****, comparison with the blank group, *p* < 0.0001. **(G)** Effects of different concentrations of RRE on glucose uptake in the HepG2 cells with insulin resistance (IR). Glucose uptake was characterized by fluorescence intensity of 2-NBDG. MET, Metformin. *, **, ***, ****, comparison with the model group, *p* < 0.05, *p* < 0.01, *p* < 0.001, *p* < 0.0001, respectively.

Compared to the blank group, the glucose uptake of the HepG2 cells with IR in the model group was significantly reduced (*p* < 0.05) and that of the positive control group was increased (Figure 1F; Supplementary Figure S2). Compared to the model group, RRE (25–100 μg/ml) can increase glucose uptake of the cells and inhibit IR of the cells with increasing concentration of RRE, of which the glucose uptake of the cells increases 45.76 ± 3.03% in the RRE (100 μg/ml) group (Figure 1G).

### *Rehmanniae* Radix Extract Ameliorates Diabetic Conditions in Streptozocin-Induced Diabetes Mellitus Rats

The DM-related indicators such as body weight, FBG, OGTT, and IST were determined after administrating RRE for 4 weeks (Figure 1A). The weekly weight was significantly increased and the weekly FBG was significantly decreased in the rats that of the RRE group than that of the model group during the experimental cycle (*p* < 0.05, Figures 1B, C). After the DM rats were administrated RRE for 4 weeks, their weight was 10.70 ± 2.00% higher (Figure 1B) and FBG was 73.23 ± 3.33% lower (Figure 1C) than those of the model group. For OGTT, the AUC for blood glucose was 12.31 ± 2.29% lower in the rats of the RRE group than in those of the model group (Figure 1D). For IST, the AUC for blood glucose was 13.61 ± 5.60% lower in the rats of the RRE group than in those of the model group (Figure 1E). All these results indicated that RRE can alleviate DM.

### Prediction of Antidiabetic Effect of *Rehmanniae* Radix Extract Based on Network Pharmacology Analysis

Seventy-six components in the RR were screened using the TCMSP database. According to the screening conditions, 17 components were obtained. At the same time, the 76 components were screened using the SwissADME database and 24 of them met the requirements. Thirty-two components were obtained by combining the screened components based on the TCMSP and Swiss databases. One hundred twenty-eight drug targets with 18 components were obtained by combining the TCMSP database and the SwissTargetPrediction database, and 60 targets of these were non-repetitive (Supplementary Table S1).

**FIGURE 2:**
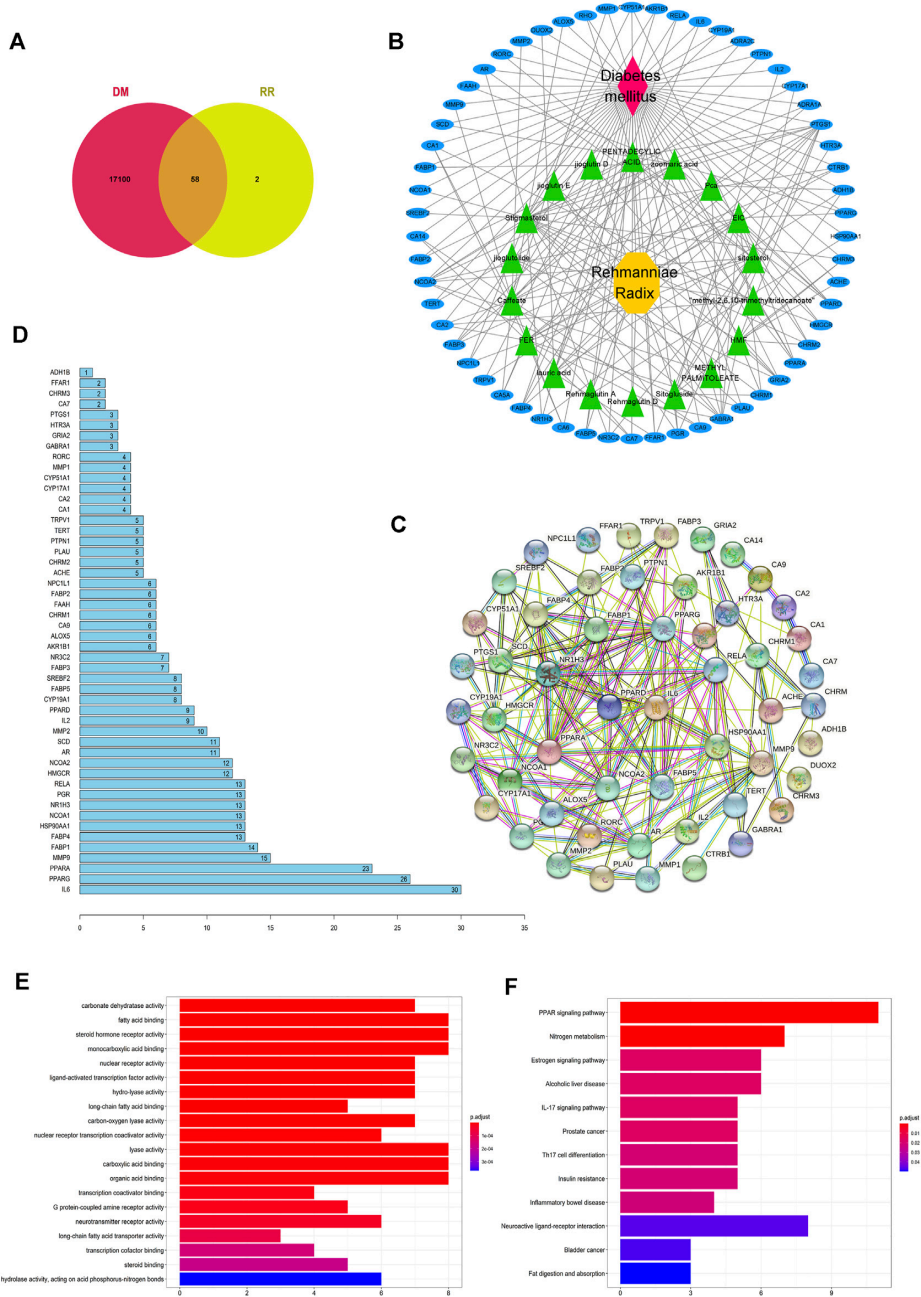
Potential antidiabetic targets of RR were predicted by network pharmacology. **(A)** Venn diagram of the intersection of the targets between RR and DM. **(B)** RR–components–targets–DM network. For the nodes, red represents DM, yellow represents RR, green represents components of RR, and blue represents targets. Edges represent interactions. **(C)** Diagram of the protein–protein interaction (PPI) network. Each node represents a protein, with the protein structure located in the middle of nodes, and edges represent interactions between nodes. **(D)** Histogram of the number of adjoining nodes in the PPI network. The number of adjoining nodes for each gene was calculated and is marked to the right of the histogram. **(E)** Histogram of GO enrichment. The abscissa represents the number of genes enriched in the GO term. The ordinate represents different GO terms, and the color represents the *p* value. Twenty GO terms with the smallest *p* value are listed in the histogram. **(F)** Histogram of KEGG enrichment. The abscissa represents the number of genes enriched in the KEGG pathway. The ordinate represents the different KEGG pathways, and the color represents different *p* value.

A total of 17,158 diabetes-related targets were screened by the GeneCards database. Fifty-eight targets were obtained by intersecting the 17,158 targets with 60 drug targets of RR (Figure 2A). Then, the RR–components–targets–DM network was established, which consisted of 18 components and 58 targets with edges connecting drugs with diseases (Figure 2B). From the network, the relationship between the active component and targets was disclosed. The target’ PPI network was established to evaluate the significance of different targets, which contained 53 nodes and 208 edges (Figure 2C). There were 16 nodes with more than 10 edges, of which IL6 ranked first from sorting the number of edges connected to each node (Figure 2D). The family members of the peroxisome proliferators–activated receptors (PPAR), fatty acid binding protein (FABP), matrix metalloproteinase (MMP), and nuclear receptor corepressor (NCOA) occupy the majority. They play important roles in the fight against DM using RR.

Of the GO enrichment analysis of 58 targets, 6 significantly enriched GO terms are DM-related, namely, regulation of the fatty acid binding (GO:0005504), steroid hormone receptor activity (GO:0003707), monocarboxylic acid binding (GO:0033293), lyase activity (GO:0016829), carboxylic acid binding (GO:0031406), and organic acid binding (GO:0043177) (Figure 2E). Besides, many GO terms are related to lipid metabolism, which implies these pathways are important for RR against DM.

According to the screening conditions of p.adjust < 0.1 in the KEGG enrichment analysis, 12 pathways were obtained (Figure 2F). Among them, the PPAR signaling pathway (hsa03320) ranked first in terms of *p* value and gene count. It indicated that PPAR was one of the important antidiabetic signaling pathways of RR. RR possibly influences the PPAR pathway by affecting its genes (i.e., *PPAR*, *SCD1*, *MMP*, and *FABP*). It is worth mentioning that neuroactive ligand–receptor interaction (hsa:04080) appeared in the enriched KEGG pathways and the gene count (e.g., *TRPV1*) ranked second.

### Identification of Antidiabetic Mechanism of *Rehmanniae* Radix Extract by Transcriptomics Analysis

In addition to network pharmacology, we also used the transcriptomics approach to further clarify the potential mechanisms of RRE against DM. The raw data have been deposited in the SRA database of NCBI and the accession number is PRJNA807859.

In the transcriptome analysis of the kidneys, a total of 1,205 DEGs were identified in the control *vs*. model group, 509 of which were upregulated and 696 were downregulated. Compared to the model group, a total of 1,033 DEGs were identified in the RRE group, 474 of which were upregulated and 559 were downregulated.

**FIGURE 3:**
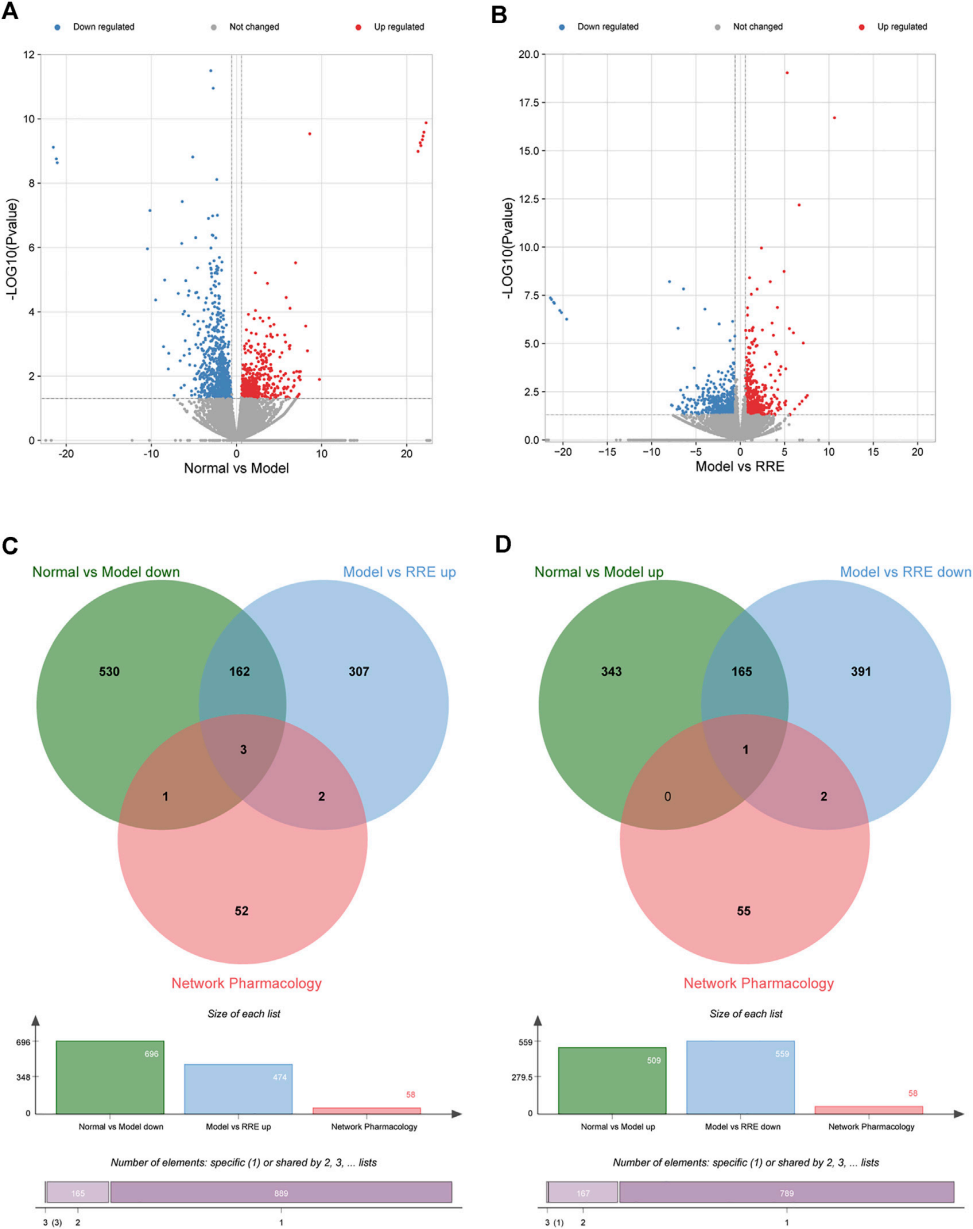
Potential antidiabetic targets of RR were predicted by transcriptome analysis. **(A)** Volcano map shows the number of DEGs in the normal *vs*. model group; **(B)** Volcano map shows the number of DEGs in the model *vs*. RRE group. The abscissa represents the fold change of the gene, and the ordinate represents the *p* value. Each dot in the figure represents a specific gene, the red and blue dots represent significantly upregulated and downregulated genes, respectively, while the gray dots represent nonsignificantly different genes. Screening criteria are |FC| >1.5 and *p* < 0.05. **(C,D)** Venn diagrams: the circles in different colors in the Venn diagram represent a DEGs set and the intersection of the circles represents the number of DEGs shared between each set. **(C)** Intersection of DEGs from the normal *vs*. model group (down) and the model *vs*. RRE group (up) by transcriptomics and network pharmacology analysis. **(D)** Intersection of DEGs from the normal *vs*. model group (up) and the model *vs*. RRE group (down) by transcriptomics and network pharmacology analysis.

**FIGURE 4:**
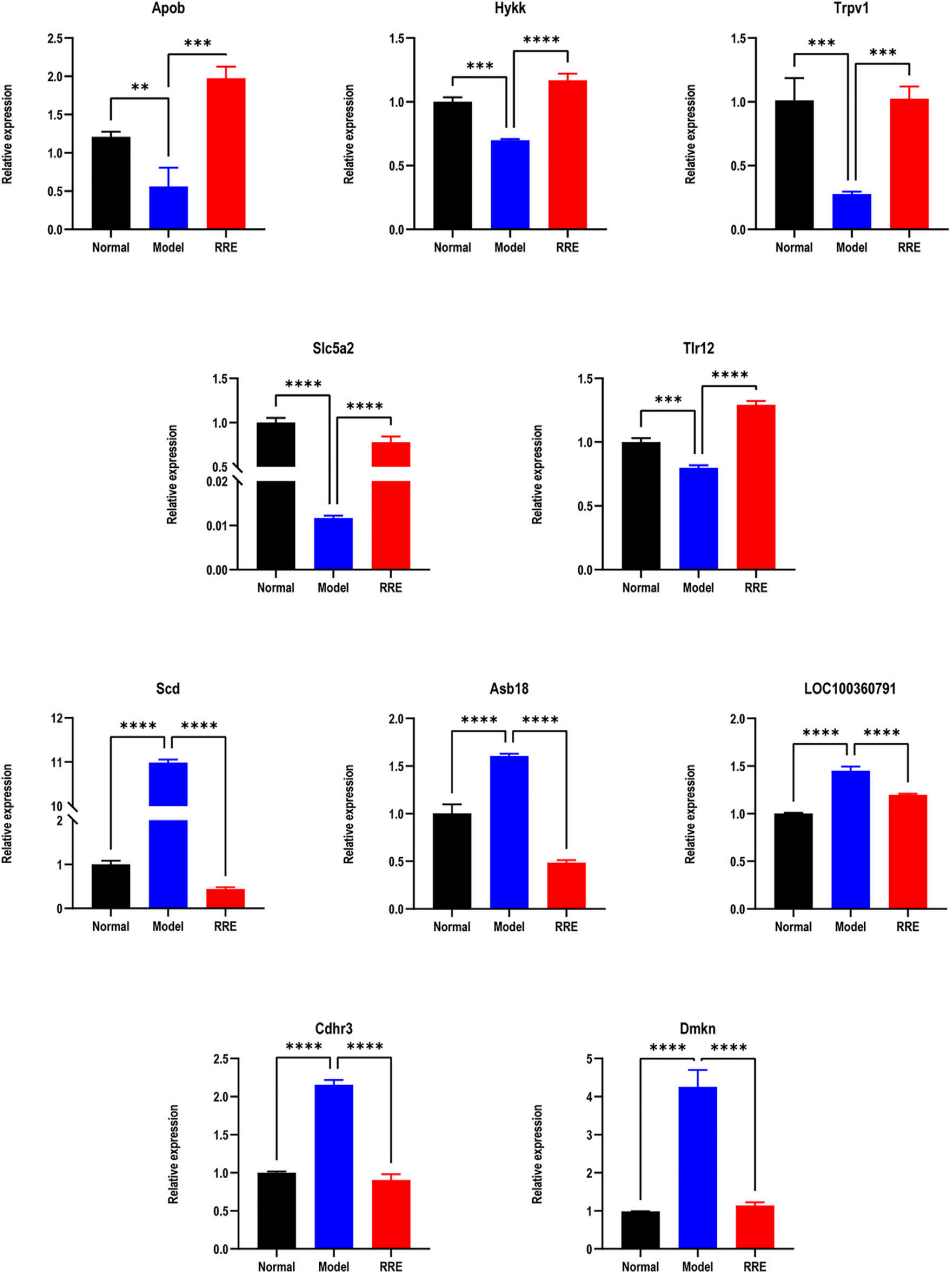
The expression tendency of DEGs by qRT-PCR. Relative expressions of *Apob*, *Hykk*, *Trpv1*, *Slc5a2*, and *Tlr12* in the normal *vs*. model group (down) and the model *vs*. RRE group (up). Relative expression of *SCD1*, *Asb18*, *LOC100360791*, *Cdhr3*, and *Dmkn* in the normal *vs*. model group (up) and the model *vs.* RRE group (down)*.*

From the volcano diagram, the fold change of DEGs and their statistical *p* values are intuitively seen (Figures 3A, B). In order to clarify the relationship among the DEGs, DM, and RRE, their interactions were analyzed. The results indicated that there were 165 DEGs between the normal *vs*. model group (downregulation) and the model *vs*. RRE group (upregulation) (Figure 3C) and that there were 166 DEGs between the normal *vs*. model group (upregulation) and the model *vs*. RRE group (downregulation) (Figure 3D), which are an important reference for us to mine DM-related target genes. The DEGs’ expression trends by qRT-PCR were mostly consistent with that of the transcriptome analysis (Figure 4).

**FIGURE 5:**
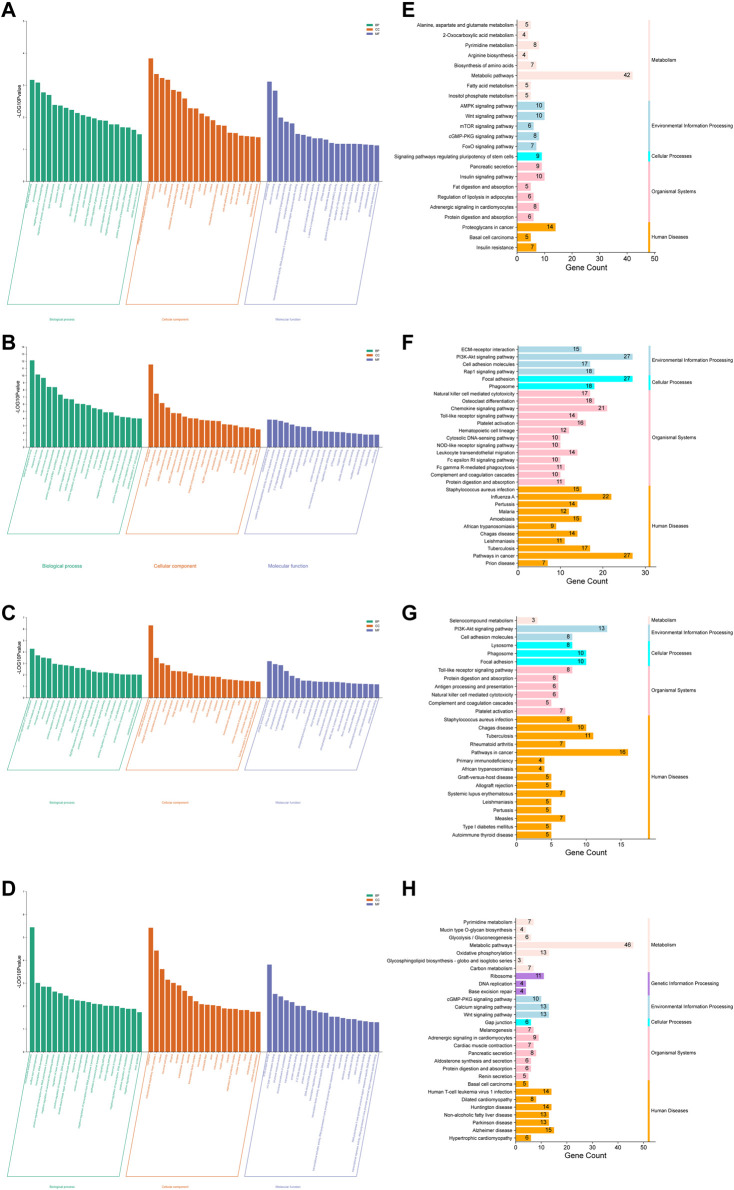
Potential antidiabetic pathways of RR were enriched by the GO and KEGG analyses. **(A–D)** Histogram of GO enrichment analysis: **(A)** the normal *vs*. model group (up); **(B)** the normal *vs*. model group (down); **(C)** the model *vs*. RRE group (up); **(D)** the model *vs*. RRE group (down). The abscissa represents the different GO terms belonging to the three categories, namely, biological process, cellular component, and molecular function. The ordinate represents the logarithmized *p* value (the enrichment result with the 20 minimum *p* values are displayed). **(E–H)** Histogram of KEGG enrichment analysis: **(E)** the normal *vs*. model group (up); **(F)** the normal *vs*. model group (down); **(G)** the model *vs*. RRE group (up); **(H)** the model *vs*. RRE group (down). The abscissa represents a number of genes enriched in this pathway. The left ordinate represents the KEGG pathways, the right ordinate represents the categories of the pathways.

DEGs were enriched by the GO database. The results indicated that 128 GO terms were upregulated (Figure 5A) and 314 GO terms were downregulated (Figure 5B) in the normal *vs*. model group. Of them, the potential DM-related signal pathways included adipocyte differentiation (e.g., brown fat cell differentiation and positive regulation of fat cell differentiation), lipid synthesis and metabolism (e.g., fatty acid homeostasis, lipid catabolic process, and lipid metabolic process), glycogen synthesis and metabolism (e.g., gluconeogenesis and glycogen biosynthetic process), apoptosis (e.g., apoptotic process and programmed necrotic cell death), oxidative stress (e.g., cellular response to oxidative stress and response to endoplasmic reticulum stress), and inflammation (e.g., chronic inflammatory response and interleukin-1 beta production). In addition, 164 GO terms were upregulated (Figure 5C) and 153 GO terms were downregulated (Figure 4D) in the model *vs*. RRE group. The DM-related signaling pathways in the model *vs*. RRE group were mostly consistent with those of the normal *vs*. model group.

The results based on the KEGG analysis indicated that 24 KEGG pathways were upregulated (Figure 5E), while 66 KEGG pathways were downregulated (Figure 4F) in the normal *vs*. model group. Among these KEGG pathways, the metabolic pathways (hso01100) included the largest number of DEGs. In addition, KEGG pathways such as apoptosis, inflammation, glycolipid metabolism, and pancreatic secretion were consistent with that of GO enrichment terms. The AMPK, cGMP-PKG, RAS, and FoxO signaling pathways as well as IR were also listed in the KEGG pathways. Similarly, 27 KEGG pathways were upregulated (Figure 5G), while 29 KEGG pathways were downregulated (Figure 5H) in the model *vs*. RRE group. KEGG pathways such as toll-like receptor, PI3K-Akt, cGMP-PKG, calcium signaling pathway, and oxidative phosphorylation were consistent with that of the GO enrichment terms.

Finally, four collective targets were discovered by the intersection of network pharmacology and transcriptomics analysis, namely, *TRPV1*, *FABP2*, *HSP90AA1*—in the normal *vs*. model group (downregulation) and the model *vs*. RRE group (upregulation) and *SCD1*—in the normal *vs*. model group (upregulation) and the model *vs*. RRE group (downregulation). Among the four targets, *TRPV1* and *SCD1* are the important targets related to DM (Enoch et al., 1976; Radu et al., 2013). *TRPV1* possibly delays the progression of DM and *SCD1* possibly accelerates the progression of DM.

Transcriptome analysis of the small intestine indicated that DEGs and the GO and KEGG pathway enrichment in the normal *vs*. model group and the model *vs*. RRE group showed upregulation and downregulation, respectively (Supplementary Figures S4, S5). The transcriptome analysis of the skeletal muscle indicated that DEGs and the GO and KEGG pathway enrichment in the normal *vs*. model group and the model *vs*. RRE group also showed upregulation and downregulation, respectively (Supplementary Figures S6, S7). However, the intersection analysis by the transcriptome analysis and network pharmacology indicated that there is only one intersection target in the small intestine and no intersection target in the skeletal muscle (Supplementary Figures S4, S6). This suggests that the kidney is the main target organ for the antidiabetic effect of RR.

### Overexpressions of TRPV1 and SCD1 Affects Cell Apoptosis and Oxidative Stress

**FIGURE 6:**
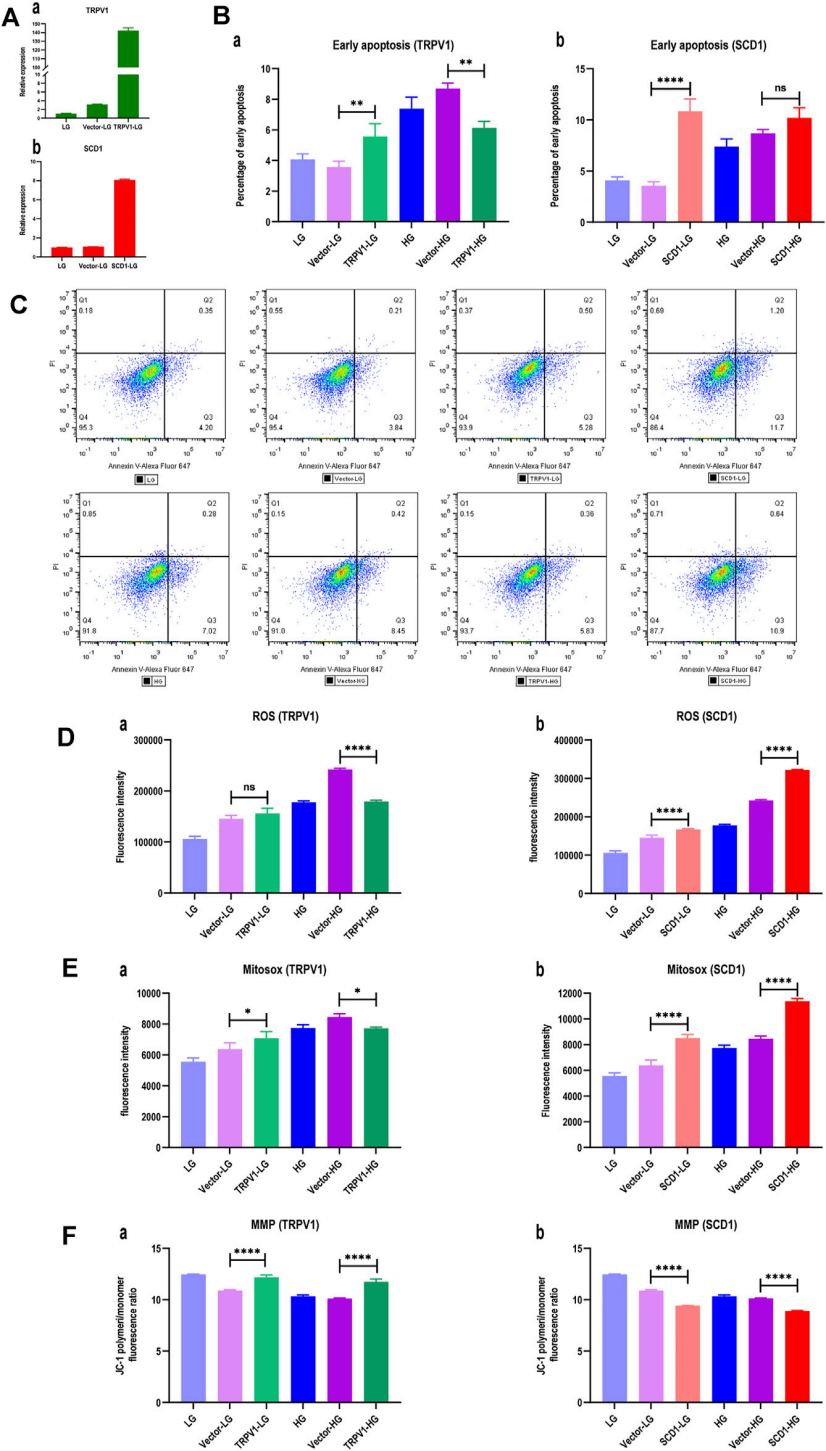
Overexpressions of TRPV1 and SCD1 in HK2 cells: LG, low glucose; Vector-LG, vector plasmid transfected low glucose; TRPV1-LG, TRPV1 plasmid transfected low glucose; SCD1-LG, SCD1 plasmid transfected low glucose; HG, high glucose; Vector-HG, vector plasmid transfected high glucose; TRPV1-HG, TRPV1 plasmid transfected high glucose; SCD1-HG, SCD1 plasmid transfected high glucose. **(A)** Gene expression after transfecting *TRPV1* and *SCD1*: (a) gene expression of *TRPV1*; (b) gene expression of *SCD1*. **(B)** Histogram of percentage of early apoptosis of HK2 cells: (a) percentage of early apoptosis of HK2 cells with overexpression of *TRPV1*; (b) percentage of early apoptosis of HK2 cells with overexpression of *SCD1*. ns, no statistical difference; **, *p* < 0.01; ***, *p* < 0.001. **(C)** Dot plot of flow cytometry for apoptosis detection of HK2 cells, which are gated based on the compensation and positive reference. The ratio of Q3 to live cells represents the percentage of early apoptosis. **(D)** Histogram of intracellular reactive oxygen species (ROS) of HK2 cells: (a) ROS in HK2 cells with overexpression of *TRPV1*; (b) ROS in HK2 cells with overexpression of *SCD1*. ns, no statistical difference; ****, *p* < 0.0001. **(E)** Histogram of mitochondrial superoxide (MitoSOX) of HK2 cells: (a) MitoSOX in HK2 cells with overexpression of *TRPV1*; (b) MitoSOX in HK2 cells with overexpression of *SCD1*. *, *p* <0.05; ****, *p* <0.0001. **(F)** Histogram of mitochondrial membrane potential (MMP) of HK2 cells transfected *TRPV1* and *SCD1*, respectively: (a) MMP in HK2 cells with overexpression of *TRPV1*; (b) MMP in HK2 cells with overexpression of *SCD1*. ns, no statistical difference; ****, *p* < 0.0001.

In order to verify the roles of TRPV1 and SCD1 in the treatment of DM, the HK2 cells overexpressed *TRPV1* or *SCD1* were constructed and verified by the qRT-PCR (Figure 6A). The detection of cell apoptosis indicated that early apoptosis of HG group was 1.75 times of that of the LG group, indicating that high glucose significantly induced apoptosis of HK2 cells. Compared to the vector-LG group, the apoptosis ratio of the TRPV1-LG group increased. However, the apoptosis ratio of HK2 cells was significantly lower in the TRPV1-HG group than in the vector-HG group (*p* < 0.01) (Figures 6Ba, C). The apoptosis ratio of HK2 cells was 3.05 times higher in the SCD1-LG group than in the vector-LG group (*p* < 0.0001). However, compared with the vector-HG group, the apoptosis ratio of HK2 cells in the SCD1-HG group exhibited increased tendencies in the high glucose environment (Figures 6Bb, C). Thus, it can be seen that the overexpression of *TRPV1* inhibits early apoptosis induced by high glucose, while the overexpression of *SCD1* can aggravate early apoptosis in the normal environment.

Compared with the vector-HG group, ROS in HK2 cells from the TRPV1-HG group decreased by 25.89% (*p* < 0.0001), which was not seen in the TRPV1-LG *vs*. vector-LG group (Figure 6Da). It indicated that the overexpression of *TRPV1* reduces the degree of oxidative stress induced by high glucose. When compared with the vector-LG group, ROS in HK2 cells from the SCD1-LG group was higher (*p* < 0.0001), and when compared with the vector-HG group, ROS in HK2 cells from the SCD1-HG group was greatly increased by 33.01% (Figure 6Db). This indicates that the overexpression of *SCD1* enhances cellular oxidative stress both in low-glucose and high-glucose environments.

When compared with the vector-HG group, MitoSOX in HK2 cells from the TRPV1-HG group was decreased (*p* < 0.05), which was not observed in the TRPV1-LG *vs*. vector-LG group (Figure 6Ea). This suggests that the overexpression of *TRPV1* reduces the degree of oxidative stress induced by HG. When compared with the vector-LG group, MitoSOX in HK2 cells in the SCD1-LG group was 33.43% higher (*p* < 0.0001) and compared with the vector-HG group, MitoSOX in HK2 cells in the SCD1-HG group was increased by 34.55% (Figure 6Eb). This suggests that the overexpression of *SCD1* enhances cellular oxidative stress both in the LG and HG environments.

The mitochondrial membrane potential (MMP) is a more sensitive indicator for oxidative stress. It was found that when compared with the low-glucose group, the MMP of HK2 cells decreased in the high-glucose group. When compared with the vector-LG group, the MMP of HK2 cells in the TRPV1-LG group was significantly elevated (*p* < 0.0001). When compared with the vector-HG group, the MMP of HK2 cells in the TRPV1-HG group increased, indicating that the overexpression of *TRPV1* decreases the influence of high glucose on MMP (Figure 6Fa). When compared with the vector-HG group, the MMP of HK2 cells in the SCD1-HG group increased. It is noteworthy that the MMP of HK2 cells increased by three-fourth folds in the SCD1-LG group when compared with the vector-LG group (Figure 6Fb). This shows that regardless of the high-glucose environment, *SCD1* is an independent risk factor that affects MMP.

### mRNA and Protein Expressions of TRPV1 in the HK2 Cell Were Significantly Increased in the Presence of *Rehmanniae* Radix Extract

**FIGURE 7:**
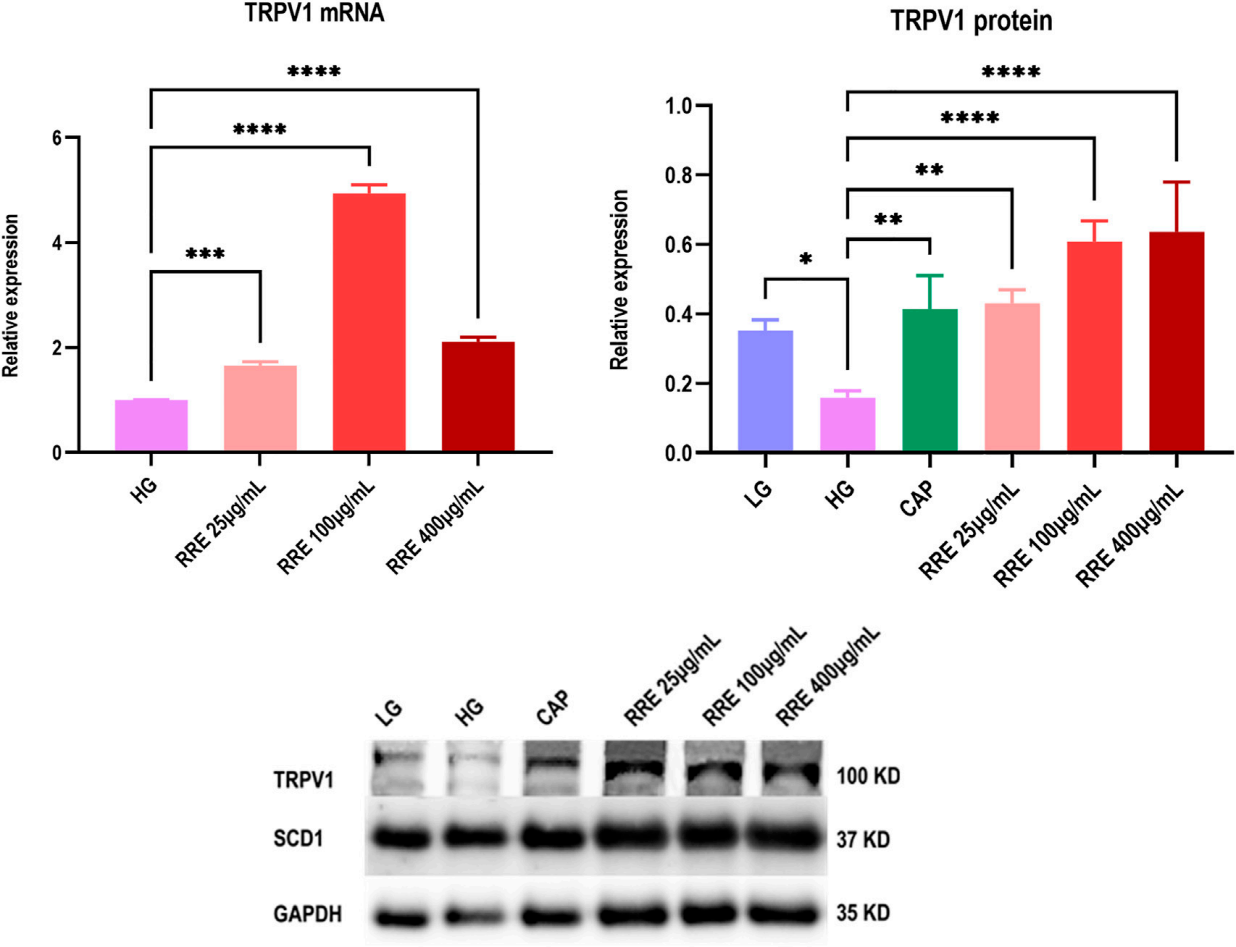
Effect of RRE on mRNA and protein expressions of TRPV1 in HK2 cells: CAP, capsaicin; TRPV1, transient receptor potential vanilloid 1; LG, low glucose; HG, high glucose. Comparison with the HG group, *, *p*<0.05; **, *p*<0.01; ***, *p*<0.001; ****, *p*<0.0001.

The assay of qRT-PCR (Figure 7) showed that the mRNA expression of TRPV1 was significantly increased in the presence of RRE (25–400 μg/ml) in the HG group. It had increased by 3.94 ± 0.08 folds when the RRE concentration was 100 μg/ml.

The assay of western blot (Figure 7) showed that the TRPV1 protein expression was significantly reduced in the HG group when compared with the LG group. The reducing trend of the protein expression of TRPV1 reversed in the presence of RRE (25–400 μg/ml) in the HG environment and increased by 2.83 ± 0.40 folds when the RRE concentration was 100 μg/ml. This suggests that RRE demonstrates significant antidiabetic effects by upregulating the mRNA and protein expressions of TRPV1 in the diabetic state. It needs to be noted that there was no significant effect on the expressions of both mRNA and protein of SCD1 in the presence of RRE (25–400 μg/ml).

## Discussion

In order to systematically and comprehensively disclose the antidiabetic mechanism of herbs (namely, RR), we integrated the methods of network pharmacology and transcriptomics. A total of 18 components of RR and 58 DM-related targets were found by network pharmacology and 331 targets were screened by transcriptomics. Finally, we screened out two important DM-related targets, namely, *TRPV1* and *SCD1*.

As a hot drug target, TRPV1 was discovered in 1997 by David Julius. He won the 2021 Nobel Prize for the research on TRPV1. At present, studies of TRPV1 focus on insulin release, peripheral IR, and renal blood vessels. TRPV1 receptors are mainly distributed in the sensory nerve endings and are also widely expressed in many tissues which are closely related to DM, such as the pancreatic islets, liver, gastrointestinal epithelium, and adipose tissue (Suri and Szallasi, 2008). In recent years, scientists have found TRPV1 plays an important role in metabolic diseases (Radu et al., 2013) and is associated with many signaling pathways such as pancreatic secretion, glucagon signaling, inflammatory mediator regulation of TRP channels, IR, and AMPK (Pabbidi et al., 2008; Sadeh et al., 2013). These signaling pathways are also enriched in our transcriptome analysis.

Activation of TRPV1 leads to a decrease in adipose accumulation and an increase in energy expenditure, which is closely linked to the activation of the PPAR and AMPK signal pathways (Brondani et al., 2012; Baskaran et al., 2016). At present, there are fewer studies on the roles played by TRPV1 in renal glycolipid metabolism. The experimental results of HK2 cells indicate that TRPV1 was upregulated (i.e., activated) by RRE, which possibly affected the PPAR and AMPK pathways and finally affected the treatment of DM.

From the results of the protein–protein interaction, it is clear that the PPAR family had a number of high ranking genes, covering *PPARA*/*PPARD*/*PPARG*. In the GO analysis, *PPARs* also appear in fatty acid binding, the steroid hormone receptor activity, the nuclear receptor activity, and the carboxylic acid binding pathways. The PPAR signaling pathway with key targets (e.g., *SCD*, *PPAR*, *FABP*, and *MMP*) is also ranked first in terms of the number of genes based on KEGG analyses (Supplementary Figure S3). PPARα plays a key role in the transcriptional regulation of lipoprotein metabolism, affecting the production and catabolism of TG-rich lipoproteins, HDL synthesis, and the *β*-oxidation pathway (Lefebvre et al., 2006). Activation of PPARα can regulate glucose homeostasis, inhibit inflammation and thrombosis, and improve vascular function (Gross et al., 2017; Bougarne et al., 2018). PPARα expresses in glomerular and tubular cells and regulates renal lipid deposition (Braissant et al., 1996). It is reported that selective agonists of PPARα can inhibit renal tubular lipid deposition and reduce oxidative stress (Maki et al., 2017). Hebbachi et al. (2008) found that insulin-sensitive changes in the gene expression of *SCD1* required intact PPARα to mediate.

*SCD* is one of the key DM-related genes, which is an adipogenic gene and regulated by several upstream genes in the PPAR signaling pathway. Our study found that high *SCD* expressions in the kidneys of STZ-induced diabetic rats, which is consistent with previous studies (Ntambi et al., 2002; Shiwaku et al., 2004; Chow et al., 2013), which were involved in the pathogenic process of DM. After DM rats were treated with RR, the expression levels of *SCD1* decreased in the body, indicating that RR directly or indirectly regulates the expression or function of *SCD1*, thus delaying DM progression.

It should be noted that RRE displayed no significant regulation on the expressions of mRNA and protein of SCD1 on the HK2 cell, although the rat transcriptome showed a downregulation of SCD1 by RRE. We speculate that the expression of SCD1 is possibly regulated by the metabolites of RRE *in vivo*. It is possible that the HK2 cells lack the enzyme that metabolizes RRE and the metabolites of RRE cannot be produced, which leads to the regulation of SCD1 that could not occur in the *in vitro* experiments.

SCD1 is a rate-limiting enzyme that converts saturated fatty acids (SFA) to monounsaturated fatty acids (MUFA). *SCD1* is selectively expressed in the proximal tubular cells in the kidneys (Zheng et al., 2001). Many studies have shown that SCD1 plays an important role in metabolic abnormalities and regulates insulin sensitivity and controls lipid metabolism (Liew et al., 2004; Popeijus et al., 2008; Sampath and Ntambi, 2011; Liu et al., 2013). This suggests that SCD1 may be a therapeutic target for DM, obesity, hypertension, and dyslipidemia (Enoch et al., 1976). *SCD1* expression is regulated by a variety of hormonal and nutritional factors (Ntambi, 1992; Waters and Ntambi, 1994; Ntambi, 1995; Waters and Ntambi, 1996). It is positively regulated by sterol regulatory element-binding protein 1c (SREBP-1c), carbohydrate response element-binding protein (CHREBP), and liver X receptor (LXR). LXR and SREBP-1c play an important role in regulating insulin-mediated lipid synthesis, while CHREBP mediates glucose-induced lipid synthesis. Thus, the activation of PPARα, LXR, and SREBP-1c, and their regulation of SCD1 are potential pathways in metabolic diseases, but the mechanisms of action in some tissues (e.g., the kidneys) have not yet been elucidated. Our study suggests that the PPAR pathway is indispensable for the pathogenesis and treatment of DM by RRE, which in turn acts to regulate the activity of SCD1 *via* multiple pathways.

TRPV1 and SCD1 play important roles in the treatment of DM. Page et al. (2019) reported that the *SCD1* expression was greatly increased in *TRPV1* knockout mice than in the wild-type mice. It implies that SCD1 activity possibly reduces when *TRPV1* is induced, which is a benefit for the treatment of DM. It is consistent with the findings of our study. A study suggested that TPRV1 regulates the expression of *SCD1* and the endocannabinoid system [e.g., cannabinoid receptor 1 (CB1)]. Meanwhile, activation of CB1 subsequently affects TRPV1 activity and enhances the *SCD1* expression (Hermann et al., 2003; Tóth et al., 2009; Liu et al., 2013). This suggests that there is an interaction between TRPV1 and SCD1 such that we can employ it against DM.

**FIGURE 8:**
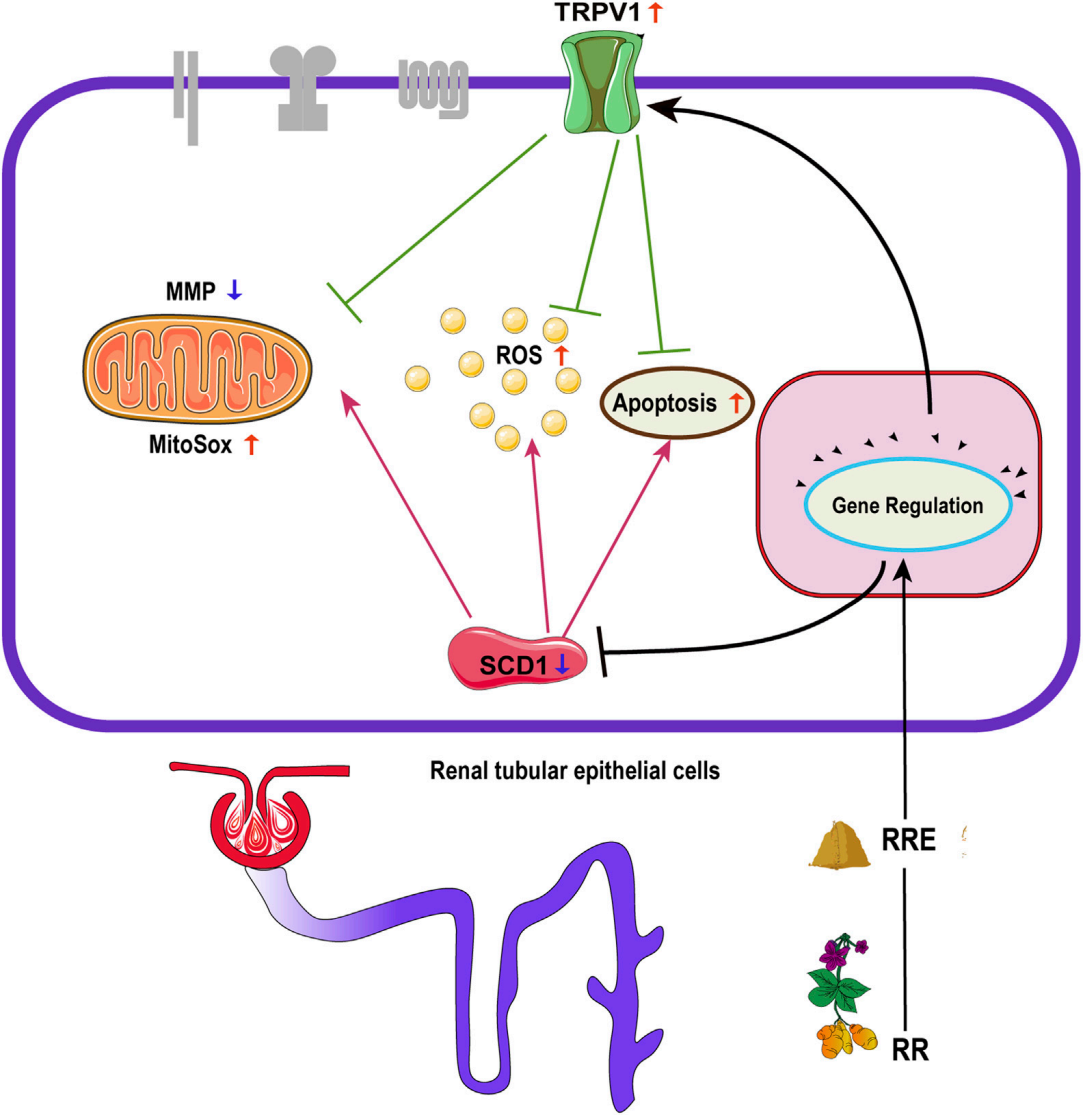
Effect of regulation of TRPV1 and SCD1 by RR on apoptosis, ROS, MitoSOX, and MMP: RR, *Rehmanniae* Radix; RRE, *Rehmanniae* Radix extract; TRPV1, transient receptor potential vanilloid 1; SCD1, stearoyl-CoA desaturase 1; ROS, reactive oxygen species; MitoSOX, mitochondrial superoxide; MMP, mitochondrial membrane potential.

Apoptosis, oxidative stress (e.g., generation of ROS and reduction of MitoSOX and MMP) of cells are important indicators for exploring the mechanisms of the pathogenesis and treatment of DM (Tan et al., 2007; Kim et al., 2011; Habib, 2013; Adelusi et al., 2020). Previous studies have shown that TRPV1 can inhibit endothelial and neuronal apoptosis when activated (Dai et al., 2008). While inhibition of SCD1 can promote oxidative stress and apoptosis of tumor cells (Stambolic and Woodgett, 2006; Hess et al., 2010; Minville-Walz et al., 2010). This suggests that TRPV1 and SCD1 exhibit different effects on apoptosis and oxidative stress in different cells. However, our study found that high glucose increased the proportion of apoptosis and oxidative stress (generation of ROS and MitoSOX, and decrease of MMP) in HK-2 cells. The overexpression of *TRPV1* significantly attenuated apoptosis and oxidative stress induced by high glucose, while the overexpression of *SCD1* enhanced apoptosis and oxidative stress induced by high glucose on HK2 cells (Figure 8).

## Conclusion

In summary, the key antidiabetic targets (e.g., TRPV1 and SCD1) and signaling pathways (PPAR) of RR were disclosed by network pharmacology and transcriptome analysis as well as by *in vitro* cellular experiments. The overexpression of TRPV1 can significantly inhibit cell apoptosis and oxidative stress induced by HG, while the overexpression of SCD1 is a risk factor for the enhancement of cell apoptosis and oxidative stress. RR displayed an inspiring antidiabetic effect by improving weight loss, reducing blood glucose, and improving IR, especially in upregulating the mRNA and protein expressions of TRPV1 and downregulating the mRNA expression of SCD1. TRPV1 and SCD1 are the potential antidiabetic targets of RR. It is of great importance to screen out the inducer of TRPV1 and inhibitors of SCD1 from herbs (e.g., RR) and drugs as potential antidiabetic drugs.

## Data Availability

The datasets presented in this study can be found in online repositories. The names of the repository/repositories and accession number(s) can be found below: NCBI SRA; PRJNA807859.
