# Supplementary figures and images for: Antidiabetic Effect of Rehmanniae Radix Based on Regulation of TRPV1 and SCD1
Source: Front Pharmacol. 2022 May 26;13:875014. doi: 10.3389/fphar.2022.875014 (PMC9178243; doi:10.3389/fphar.2022.875014)

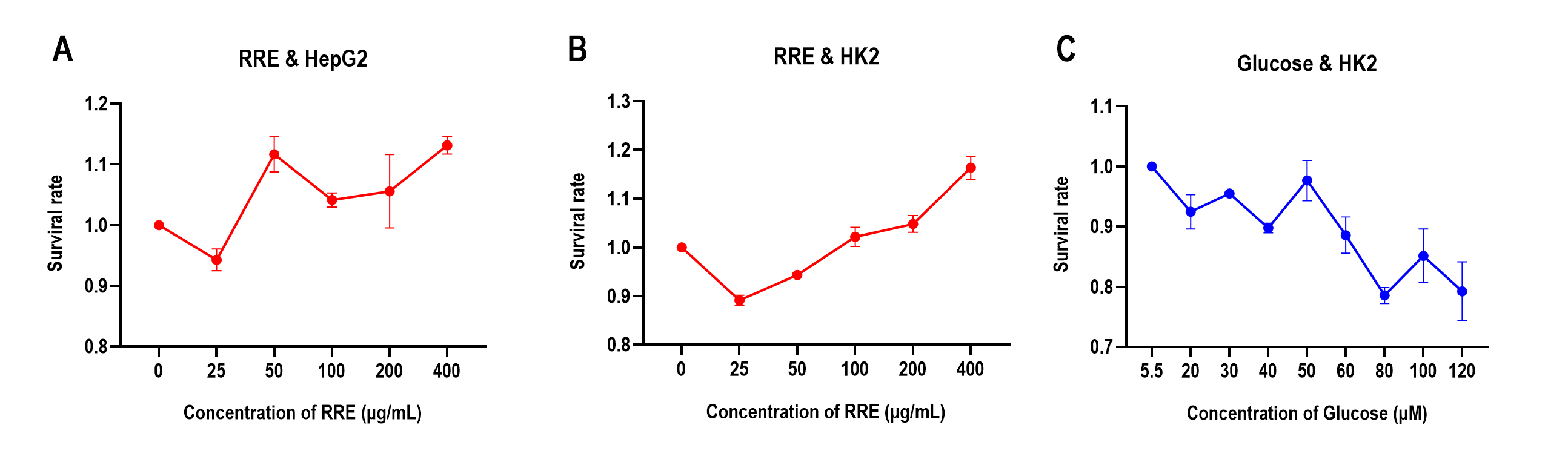

Supplement: Supplementary file 1 [file DataSheet1.ZIP › Supplementary Material Presentation/Supplementary Figure 1.tif]

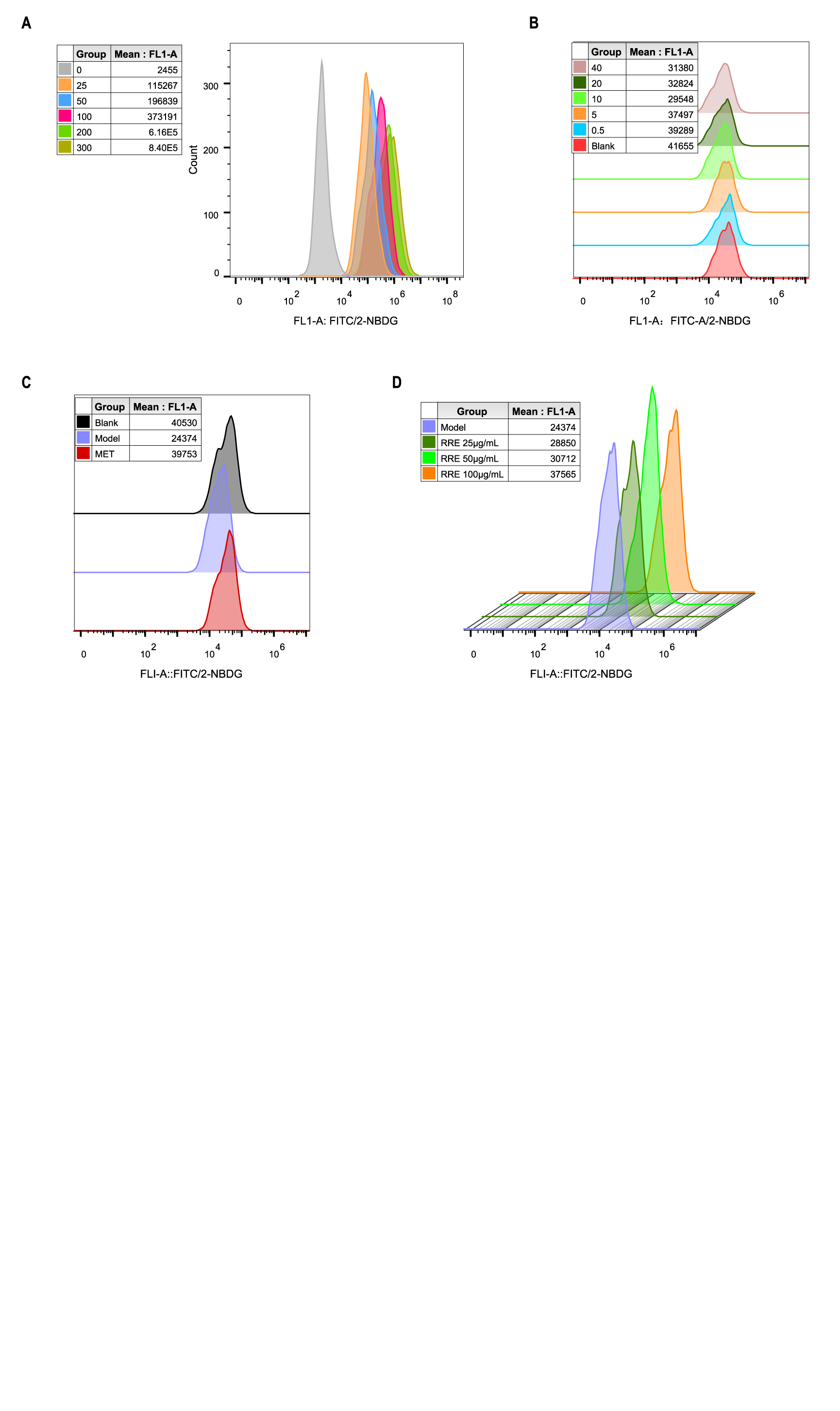

Supplement: Supplementary file 1 [file DataSheet1.ZIP › Supplementary Material Presentation/Supplementary Figure 2.tif]

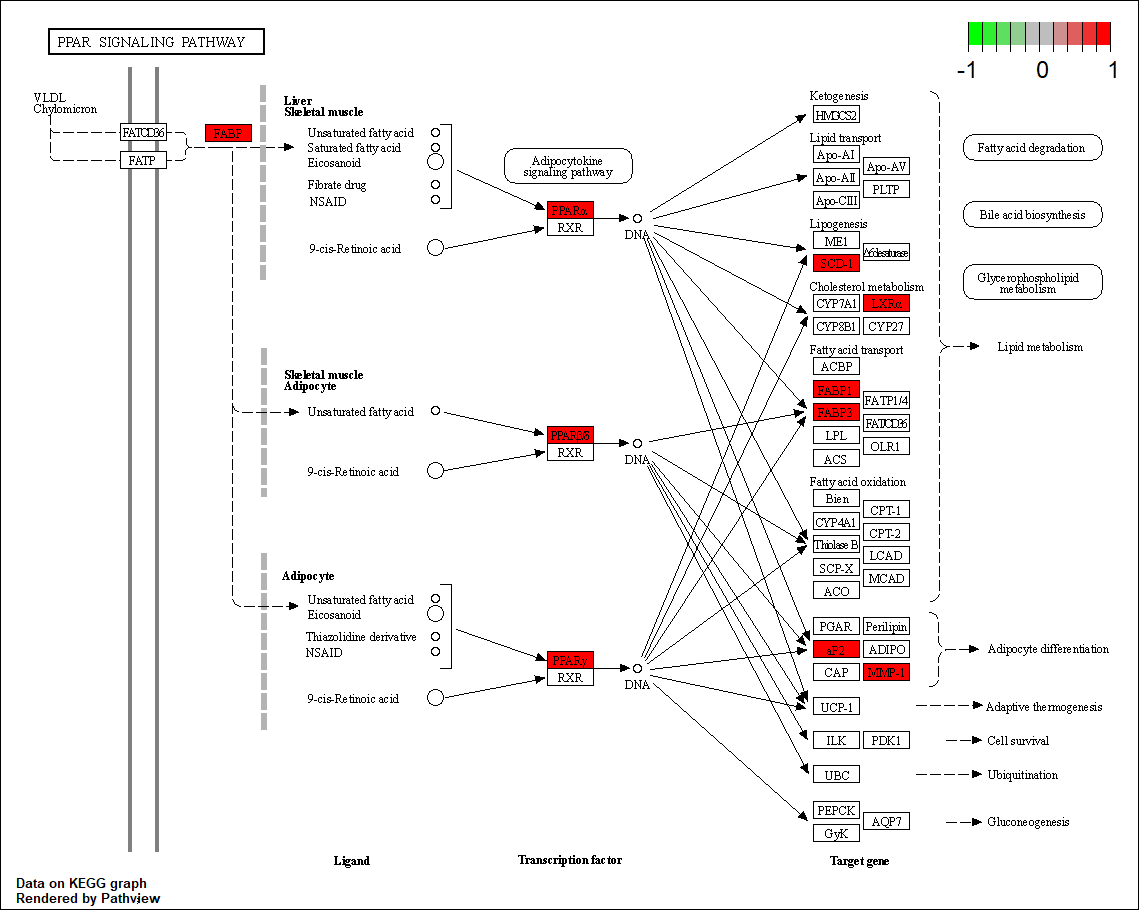

Supplement: Supplementary file 1 [file DataSheet1.ZIP › Supplementary Material Presentation/Supplementary Figure 3.png]

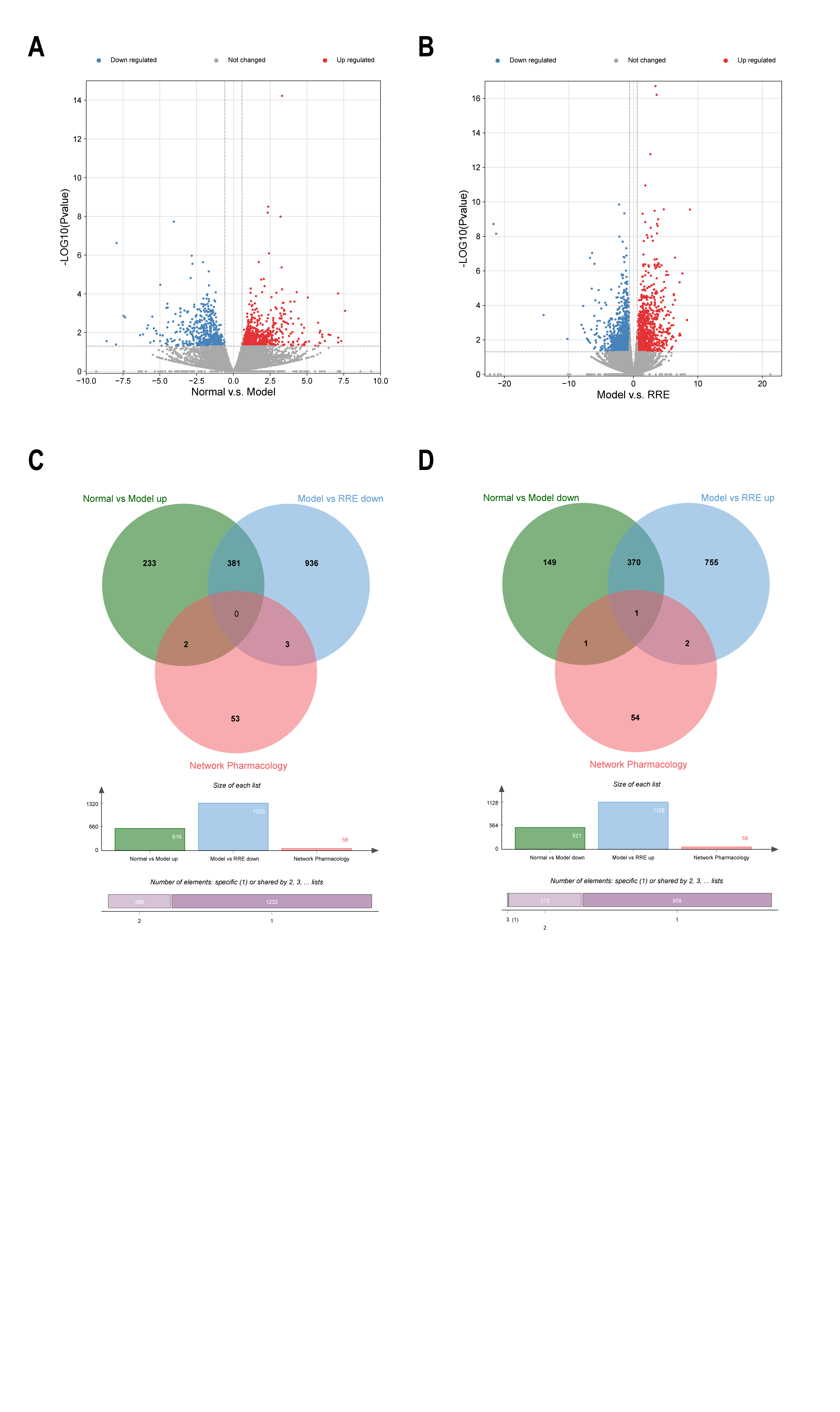

Supplement: Supplementary file 1 [file DataSheet1.ZIP › Supplementary Material Presentation/Supplementary Figure 4.tif]

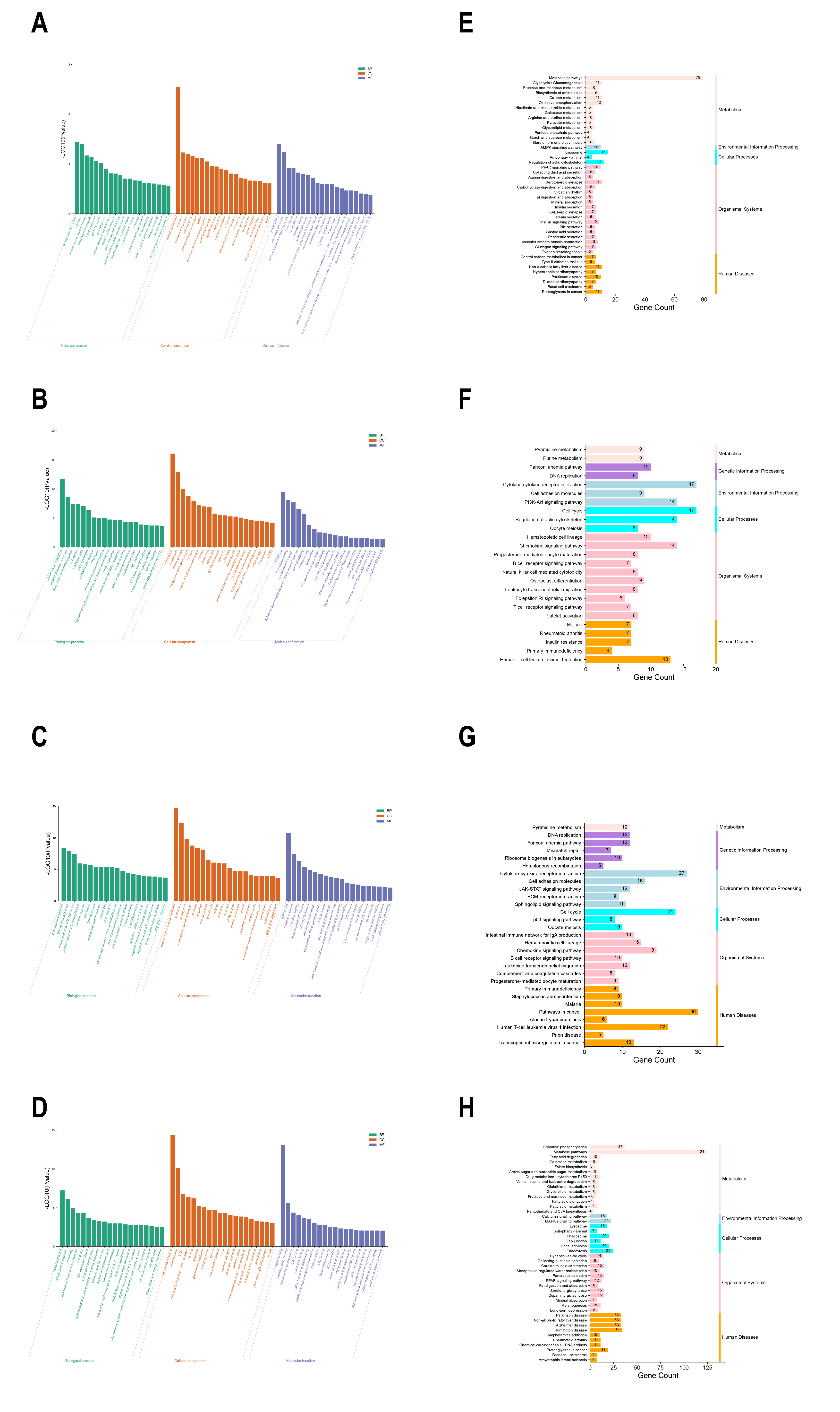

Supplement: Supplementary file 1 [file DataSheet1.ZIP › Supplementary Material Presentation/Supplementary Figure 5.tif]

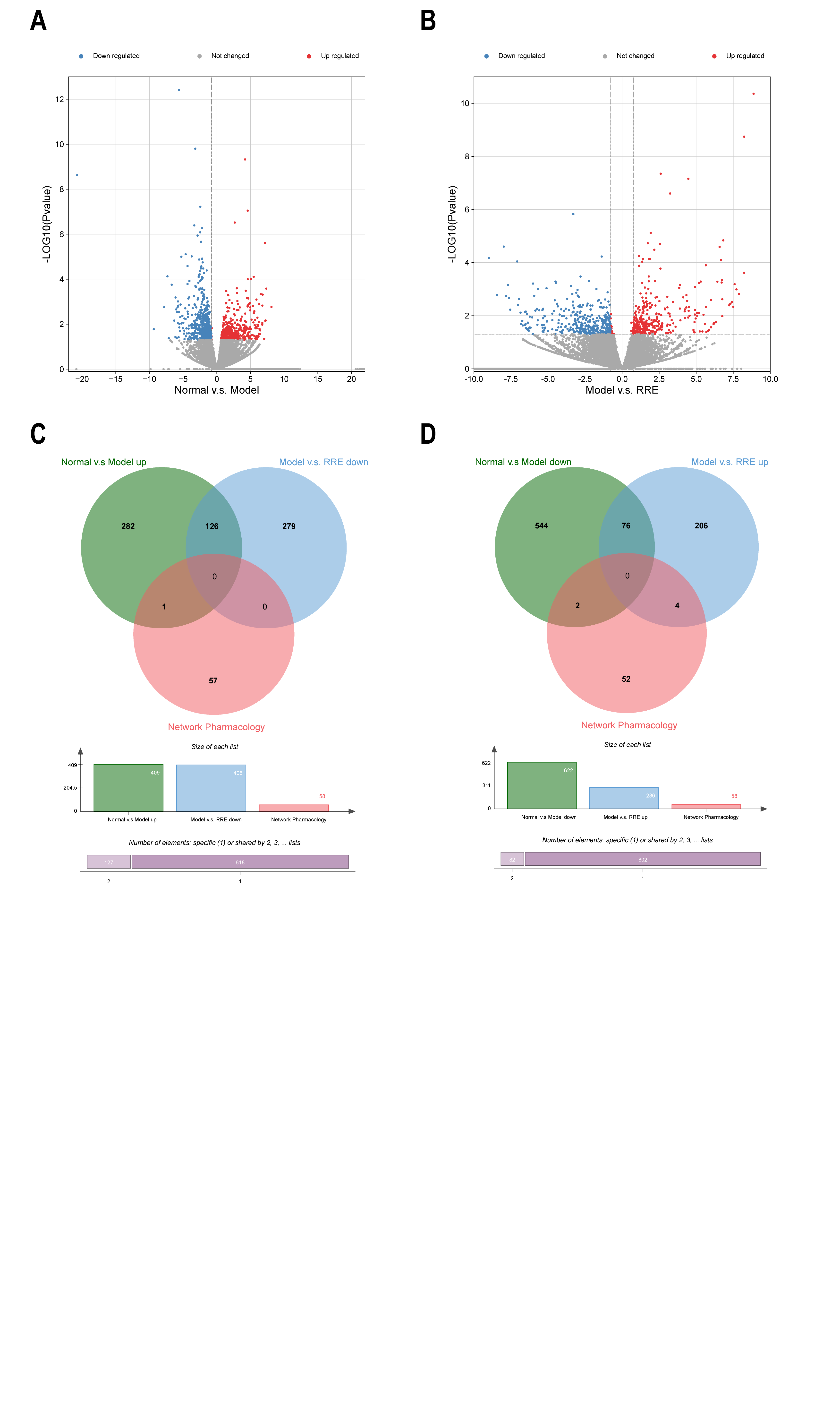

Supplement: Supplementary file 1 [file DataSheet1.ZIP › Supplementary Material Presentation/Supplementary Figure 6.tif]

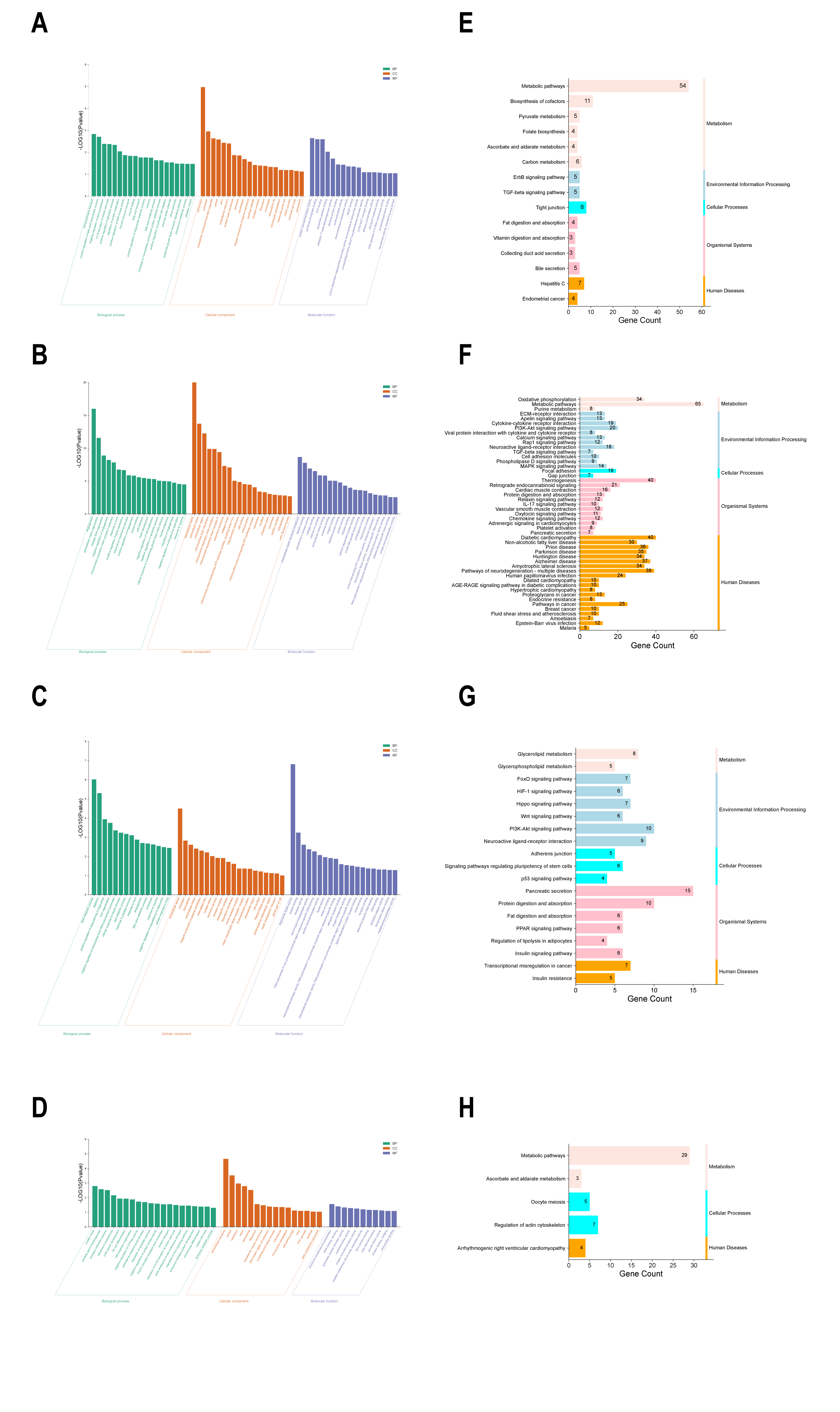

Supplement: Supplementary file 1 [file DataSheet1.ZIP › Supplementary Material Presentation/Supplementary Figure 7.tif]
