# Supplementary material for: Antidiabetic Effect of Rehmanniae Radix Based on Regulation of TRPV1 and SCD1
Source: Front Pharmacol. 2022 May 26;13:875014. doi: 10.3389/fphar.2022.875014 (PMC9178243; doi:10.3389/fphar.2022.875014)
Supplement: Supplementary file 2 [file DataSheet2.zip › Figures and supporting information.docx]

##### Supporting information

##### Supplementary Table 1 The component-target mapping table

| components | Pubchem ID | Targets | Drugs | Pubchem ID | Targets |
| --- | --- | --- | --- | --- | --- |
| Caffeate | 1549111 | *CA2* | Pca | 7405 | *GABRA1* |
| Caffeate | 1549111 | *ALOX5* | Pca | 7405 | *ADH1B* |
| Caffeate | 1549111 | *CA7* | PENTADECYLIC ACID | 13849 | *FABP4* |
| Caffeate | 1549111 | *CA1* | PENTADECYLIC ACID | 13849 | *PPARA* |
| Caffeate | 1549111 | *CA6* | PENTADECYLIC ACID | 13849 | *FABP3* |
| Caffeate | 1549111 | *MMP9* | PENTADECYLIC ACID | 13849 | *FFAR1* |
| Caffeate | 1549111 | *CA12* | PENTADECYLIC ACID | 13849 | *FABP5* |
| Caffeate | 1549111 | *MMP1* | PENTADECYLIC ACID | 13849 | *PPARD* |
| Caffeate | 1549111 | *MMP2* | PENTADECYLIC ACID | 13849 | *FABP2* |
| Caffeate | 1549111 | *PTPN1* | PENTADECYLIC ACID | 13849 | *PTGS1* |
| Caffeate | 1549111 | *CA14* | PENTADECYLIC ACID | 13849 | *RHO* |
| Caffeate | 1549111 | *CA9* | PENTADECYLIC ACID | 13849 | *NCOA2* |
| Caffeate | 1549111 | *CA5B* | Rehmaglutin A | 5320903 | *HSP90AA1* |
| Caffeate | 1549111 | *CA5A* | Rehmaglutin D | 5320906 | *GABRA1* |
| Caffeate | 1549111 | *PTGS1* | Sitogluside | 5742590 | *IL2* |
| Caffeate | 1549111 | *ADRA2C* | Sitogluside | 5742590 | *PGR* |
| Caffeate | 1549111 | *CTRB1* | Sitogluside | 5742590 | *PTGS1* |
| EIC | 5280450 | *PPARG* | Sitogluside | 5742590 | *CHRM3* |
| EIC | 5280450 | *PPARA* | Sitogluside | 5742590 | *CHRM1* |
| EIC | 5280450 | *PPARD* | Sitogluside | 5742590 | *HTR3A* |
| EIC | 5280450 | *FFAR1* | Sitogluside | 5742590 | *NCOA2* |
| EIC | 5280450 | *FABP4* | sitosterol | 12303645 | *HMGCR* |
| EIC | 5280450 | *FABP3* | sitosterol | 12303645 | *CYP51A1* |
| EIC | 5280450 | *PTGS1* | sitosterol | 12303645 | *AR* |
| EIC | 5280450 | *NCOA2* | sitosterol | 12303645 | *NPC1L1* |
| EIC | 5280450 | *GABRA1* | sitosterol | 12303645 | *NR1H3* |
| EIC | 5280450 | *TRPV1* | sitosterol | 12303645 | *CYP17A1* |
| EIC | 5280450 | *CHRM1* | sitosterol | 12303645 | *RORC* |
| EIC | 5280450 | *CHRM2* | sitosterol | 12303645 | *PGR* |
| FER | 445858 | *CA2* | sitosterol | 12303645 | *NCOA2* |
| FER | 445858 | *CA7* | sitosterol | 12303645 | *NR3C2* |
| FER | 445858 | *CA1* | Stigmasterol | 5280794 | *ADRA1A* |
| FER | 445858 | *CA6* | Stigmasterol | 5280794 | *AKR1B1* |
| FER | 445858 | *CA12* | Stigmasterol | 5280794 | *AR* |
| FER | 445858 | *CA14* | Stigmasterol | 5280794 | *CHRM1* |
| FER | 445858 | *CA9* | Stigmasterol | 5280794 | *CHRM2* |
| FER | 445858 | *CA5A* | Stigmasterol | 5280794 | *CHRM3* |
| FER | 445858 | *PTGS1* | Stigmasterol | 5280794 | *CTRB1* |
| FER | 445858 | *ADRA1A* | Stigmasterol | 5280794 | *CYP17A1* |
| FER | 445858 | *CTRB1* | Stigmasterol | 5280794 | *CYP19A1* |
| FER | 445858 | *PLAU* | Stigmasterol | 5280794 | *CYP51A1* |
| HMF | 237332 | *CHRM3* | Stigmasterol | 5280794 | *GABRA1* |
| HMF | 237332 | *CHRM1* | Stigmasterol | 5280794 | *HMGCR* |
| HMF | 237332 | *ACHE* | Stigmasterol | 5280794 | *NCOA1* |
| HMF | 237332 | *CHRM2* | Stigmasterol | 5280794 | *NCOA2* |
| HMF | 237332 | *GABRA1* | Stigmasterol | 5280794 | *NPC1L1* |
| HMF | 237332 | *ADH1B* | Stigmasterol | 5280794 | *NR1H3* |
| HMF | 237332 | *PTGS1* | Stigmasterol | 5280794 | *NR3C2* |
| HMF | 237332 | *GRIA2* | Stigmasterol | 5280794 | *PGR* |
| jioglutin D | 14486934 | *GRIA2* | Stigmasterol | 5280794 | *PLAU* |
| jioglutin E | 14486937 | *GABRA1* | Stigmasterol | 5280794 | *PTGS1* |
| jioglutin E | 14486937 | *GRIA2* | Stigmasterol | 5280794 | *SREBF2* |
| jioglutolide | 101260546 | *GABRA1* | zoomaric acid | 445638 | *PPARG* |
| jioglutolide | 101260546 | *GRIA2* | zoomaric acid | 445638 | *PPARA* |
| lauric acid | 3893 | *FFAR1* | zoomaric acid | 445638 | *PPARD* |
| lauric acid | 3893 | *PTGS1* | zoomaric acid | 445638 | *FABP4* |
| lauric acid | 3893 | *ADH1B* | zoomaric acid | 445638 | *FABP3* |
| lauric acid | 3893 | *RELA* | zoomaric acid | 445638 | *FABP5* |
| lauric acid | 3893 | *IL6* | zoomaric acid | 445638 | *FAAH* |
| lauric acid | 3893 | *DUOX2* | zoomaric acid | 445638 | *TERT* |
| METHYL PALMITOLEATE | 643801 | *PTGS1* | zoomaric acid | 445638 | *FABP1* |
| METHYL PALMITOLEATE | 643801 | *NCOA2* | zoomaric acid | 445638 | *SCD* |
| methyl-2,6,10-trimethyltridecanoate | 560276 | *CA2* | zoomaric acid | 445638 | *PTGS1* |
| methyl-2,6,10-trimethyltridecanoate | 560276 | *CA1* | zoomaric acid | 445638 | *NCOA2* |

EIC, linoleic acid; FER, ferulic acid; HMF, 5-(hydroxymethyl)furan-2-carbaldehyde;  Pca, L-Pyroglutamic acid. Supplementary Figure

##### Supplementary Table 2 Primer sequences of DEGs from the transcriptome analysis

| Gene | Forward Primer | Reverse Primer |
| --- | --- | --- |
| *Zfp449* | CCTCTCAACCCAAAGCCTCCAAAG | GTGCCCAGTAAGCTGTGACTTCC |
| *Hsp90aa1* | AACAAGCAAGACCGAACCCTCAC | CCAGCCTGCAAAGCCTCCATG |
| *Inafm1* | CGAGAGCCCAGGTGGTACGG | AGCAACACAGCAGCCAAGGATAC |
| *Anxa13* | ATGCCAAAGAGAGAAGCCATCACC | GCTGCCTCATCCGTTCCCATTC |
| *LOC108348130* | TCGCCTTCAAAGTTGTTGAC | GCAGACATCTTGGGTACCA |
| *Prss1* | CACCAGCAGCATGATTTGTG | AGCTGTCCATTGCAGACCAC |
| *LOC100909856* | ACCGCAGTACCAGTAGCCAGTAG | GGCACGCACACTGACACTGAC |
| *Cdhr3* | TCAGGTGACAGATGTGAATGAG | GCCTCCACCTGGTAAATGAA |
| *Nmrk2* | GACCGAGTGGAAGTGGTCTA | CGTACAGGTTCCCGATAGGC |
| *Dok5* | TGAGGCAGGGAGGATGTGTGAG | AGAGTGGACCTTCTGGTAGATGGC |
| *Dmkn* | GGGGGACATCTGGAGGTCAT | TCTGGTTGTTGCCTCTCACTG |
| *Asb18* | CCACCAGATTACCCCCTCAGC | GTCACCTGCCATTCCATCTCT |
| *Lce1m* | CCTCCCAAATGCAAGATCCCA | GAGAGAGAATCGGGGCTTGTG |
| *Scd* | TGTCAAAGAGAAGGGCGGAAAGC | CAGGATGAAGCACATGAGCAGGAG |
| *Hykk* | ACCGGATGGAAGCATTTACA | CATGGAGATCCCAGCTTCATAG |
| *Tlr12* | CAACCTGACGCAGACCTTGGAAG | CGGAAGCAGAGTAGACACAGAACC |
| *Ppara* | GTCATCACAGACACCCTCTC | GGACTCATCTGTACTGGTGG |
| *Stra6* | GCTATCTTGGGTTCACGTC | GAGTAGTGCTTGTAGATCTTGG |
| *Trpv1* | AGGGAGATCCATGAACCCGA | CACAGGCCGATAGTAGGCAG |
| *Apob* | GAGCCCAAGAGGAAGCATGT | GTGGACTTGGTGCTCTCGAA |
| *Pgpep1l* | GTCGGCGTGTGGCTGTTGAG | GAGAGGCAGGGTGACAGAGGAG |
| *Slc5a2* | TAACAGCAGCAGCACACTCTTCAC | AACACCACCCAAAGCCTTCCAAC |
| *Serpina12* | ACCTCACCCGCATCTCGTCTC | GCTCCTTCTGTGCCCTTCTCATTC |
| *LOC100360791* | CATGACGAGCTGTTCTCCGACATC | TGACCATCTTGCCCTCCACCTC |

##### Supplementary Table 3 Raw reads, Raw bases, Clean reads, Clean bases, Error rate (%), Q20 (%), Q30 (%), GC content (%) from transcriptome analysis

| Sample | Raw reads | Raw bases | Clean reads | Clean bases | Error rate (%) | Q20  (%) | Q30  (%) | GC content (%) |
| --- | --- | --- | --- | --- | --- | --- | --- | --- |
| Normal1 | 51565588 | 7786403788 | 46210200 | 6792478201 | 0.0259 | 97.71 | 93.25 | 43.58 |
| Normal2 | 48634502 | 7343809802 | 48142104 | 7189498112 | 0.0245 | 98.21 | 94.59 | 49.21 |
| Normal3 | 58538912 | 8839375712 | 57906684 | 8608290478 | 0.0246 | 98.19 | 94.54 | 49.22 |
| Model1 | 51067948 | 7711260148 | 44844766 | 6617184537 | 0.0248 | 98.12 | 94.37 | 50.02 |
| Model2 | 48578636 | 7335374036 | 42526474 | 6273019738 | 0.0251 | 97.98 | 94.06 | 49.56 |
| Model3 | 51753630 | 7814798130 | 45398866 | 6733097056 | 0.0245 | 98.23 | 94.65 | 52.65 |
| RRE1 | 53632636 | 8098528036 | 47010358 | 6981782333 | 0.0247 | 98.13 | 94.39 | 50.4 |
| RRE2 | 49845912 | 7526732712 | 43688504 | 6479735436 | 0.0252 | 97.96 | 93.97 | 49.95 |
| RRE3 | 50545696 | 7632400096 | 44213498 | 6579696298 | 0.026 | 97.65 | 93.19 | 50.02 |

Note: Raw reads, the total number of entries of the original sequencing data; Raw bases, the total amount of original sequencing data (i.e., the number of raw reads multiplied by the read length); Clean reads, the total number of entries of sequencing data after quality control; Clean bases, the total amount of sequencing data after quality control (i.e., the number of clean reads multiplied by the read length); Error rate (%), the average error rate of sequencing bases corresponding to the quality control data, generally below 0.1%; Q20 (%) and Q30 (%),refer to the percentage of bases with sequencing quality above 99% and 99.9% to the total bases, respectively; GC content (%), the percentage of the sum of G and C bases corresponding to the quality control data to the total bases.

## Supplementary Figures

##### Supplementary Figure 1 Survival rate of HepG2 and HK2 cells treated with different concentrations of RRE or glucose.

**(A)**, HepG2 cells were treated with 0-400 μg/mL of RRE. (**B)**, HK2 cells were treated with 0-400 μg/mL of RRE. (**C)**, HK2 cells were treated with 5.5-120 mM of glucose.

##### Supplementary Figure 2 Histogram of Flow cytometry of glucose uptake in the HepG2 cells.

**(A)**, Fluorescence intensity of 0-300 μM of 2-NBDG in the HepG2 cell. (**B)**, Fluorescence intensity of 2-NBDG at 0-40 μg/mL of insulin in the HepG2 cell. (**C)**, Validation of HepG2 cell with IR. **D**, Fluorescence intensity of 2-NBDG in the presence of 25-100 μg/mL of RRE.

##### Supplementary Figure 3 DEGs in the PPAR signaling pathway

PPAR, peroxisome proliferator-activated receptor; DEGs from transcriptome analysis were marked red.

##### Supplementary Figure 4 Potential antidiabetic targets of RR were predicted by transcriptomics analysis of small intestine of rats

(A), Volcano map showed the number of DEGs in the normal group v.s. the model group; (B), Volcano map showed the number of DEGs in the model group v.s. the RRE group. The abscissa represents the fold change of the gene, and the ordinate represents the p value. Each dot in the figure represents a specific gene, the red and blue dots represent significantly upregulated and downregulated genes, respectively, while the gray dots represent non-significantly different genes. Screening criteria are |FC| >1.5 and p < 0.05. C and D represent the Venn figures. The circles in different colors in the Venn figure represent a DEGs set, and the intersection of the circles represents the number of DEGs shared between each set. (C), Intersection of DEGs from the normal group v.s. the model group (up) and the model group v.s. the RRE group (down) by the transcriptomics and the network pharmacology analysis. (D), Intersection of DEGs from the normal group v.s. the model group (down) and the model group v.s. the RRE group (up) by the transcriptomics and the network pharmacology analysis.

##### Supplementary Figure 5 Potential antidiabetic pathways of RR were enriched by GO and KEGG analysis of small intestine of rats

A, B, C, D represent histogram of GO enrichment analysis. (**A)** represents the normal group v.s. the model group (up). (**B)** represents the normal group v.s. the model group (down). (**C)** represents the model group v.s. the RRE group (up). (**D)** represents the model group v.s. the RRE group (down). The abscissa represents the different GO terms belonging to the three categories including biological process, cellular component, molecular function. The ordinate represents the logarithmized *p* value (the enrichment result with the minimum 20 p values were displayed). E, F, G, H represent histogram of KEGG enrichment analysis. (**E)** represents the normal group v.s. the model group (up). (**F)** represents the normal group v.s. the model group (down). (**G)** represents the model group v.s. the RRE group (up). (**H)** represents the model group v.s. the RRE group (down). The abscissa represents number of genes enriched in this pathway. The left ordinate represents the KEGG pathways, the right ordinate represents categories of the pathways.

##### Supplementary Figure 6 Potential antidiabetic targets of RR were predicted by transcriptomics analysis of skeletal muscle of rats

(A), Volcano map showed the number of DEGs in the normal group v.s. the model group; (B), Volcano map showed the number of DEGs in the model group v.s. the RRE group. The abscissa represents the fold change of the gene, and the ordinate represents the p value. Each dot in the figure represents a specific gene, the red and blue dots represent significantly upregulated and downregulated genes, respectively, while the gray dots represent non-significantly different genes. Screening criteria are |FC| >1.5 and p < 0.05. C and D represent the Venn figures. The circles in different colors in the Venn figure represent a DEGs set, and the intersection of the circles represents the number of DEGs shared between each set. (C), Intersection of DEGs from the normal group v.s. the model group (up) and the model group v.s. the RRE group (down) by the transcriptomics and the network pharmacology analysis. (D), Intersection of DEGs from the normal group v.s. the model group (down) and the model group v.s. the RRE group (up) by the transcriptomics and the network pharmacology analysis.

##### Supplementary Figure 7 Potential antidiabetic pathways of RR were enriched by GO and KEGG analysis of skeletal muscle of rats

A, B, C, D represent histogram of GO enrichment analysis. (**A)** represents the normal group v.s. the model group (up). (**B)** represents the normal group v.s. the model group (down). (**C)** represents the model group v.s. the RRE group (up). (**D)** represents the model group v.s. the RRE group (down). The abscissa represents the different GO terms belonging to the three categories including biological process, cellular component, molecular function. The ordinate represents the logarithmized *p* value (the enrichment result with the minimum 20 p values were displayed). E, F, G, H represent histogram of KEGG enrichment analysis. (**E)** represents the normal group v.s. the model group (up). (**F)** represents the normal group v.s. the model group (down). (**G)** represents the model group v.s. the RRE group (up). (**H)** represents the model group v.s. the RRE group (down). The abscissa represents number of genes enriched in this pathway. The left ordinate represents the KEGG pathways, the right ordinate represents categories of the pathways.

**Supplementary Figure 1**

**Supplementary Figure 2**

**Supplementary Figure 3**

**Supplementary Figure 4**

**Supplementary Figure 5**

**Supplementary Figure 6**

**Supplementary Figure 7**
